# Deficiency of factor-inhibiting HIF creates a tumor-promoting immune microenvironment

**DOI:** 10.1073/pnas.2309957121

**Published:** 2024-02-29

**Authors:** Jingyi Ma, Khatoun Al Moussawi, Hantao Lou, Hok Fung Chan, Yihua Wang, Joseph Chadwick, Chansavath Phetsouphanh, Elizabeth A. Slee, Shan Zhong, Thomas M. Leissing, Andrew Roth, Xiao Qin, Shuo Chen, Jie Yin, Indrika Ratnayaka, Yang Hu, Pakavarin Louphrasitthiphol, Lewis Taylor, Paulo J. G. Bettencourt, Mary Muers, David R. Greaves, Helen McShane, Robert Goldin, Elizabeth J. Soilleux, Mathew L. Coleman, Peter J. Ratcliffe, Xin Lu

**Affiliations:** ^a^Ludwig Institute for Cancer Research, Nuffield Department of Medicine, University of Oxford, Oxford OX3 7DQ, United Kingdom; ^b^Ministry of Health Holdings, Singapore 099253, Singapore; ^c^Biological Sciences, Faculty of Environmental and Life Sciences, University of Southampton, Southampton SO17 1BJ, United Kingdom; ^d^The Kirby Institute, University of New South Wales, Kensington, NSW 2052, Australia; ^e^Department of Molecular Oncology, BC Cancer, Vancouver, BC V5Z 4E6, Canada; ^f^Department of Pathology and Laboratory Medicine, University of British Columbia, Vancouver, BC V6T 1Z7, Canada; ^g^Department of Computer Science, University of British Columbia, Vancouver, BC V6T 1Z4, Canada; ^h^Department of Oncology, Faculty of Medical Sciences, University College London, London WC1E 6BT, United Kingdom; ^i^Sir William Dunn School of Pathology, University of Oxford, Oxford OX1 3RE, United Kingdom; ^j^The Jenner Institute, Nuffield Department of Medicine, University of Oxford, Oxford OX3 7DQ, United Kingdom; ^k^Center for Interdisciplinary Research in Health, Faculty of Medicine, Universidade Católica Portuguesa, Lisbon 1649-023, Portugal; ^l^Department of Metabolism, Digestion and Reproduction, Faculty of Medicine, Imperial College London, London W2 1NY, United Kingdom; ^m^Department of Pathology, University of Cambridge, Cambridge CB2 1QP, United Kingdom; ^n^Institute of Cancer and Genomic Sciences, University of Birmingham, Birmingham B15 2TT, United Kingdom

**Keywords:** tumor microenvironment, hypoxia-inducible factor, factor-inhibiting HIF, B cell lymphoma, tumor suppression

## Abstract

Hypoxia signaling influences tumor development through both cell-intrinsic and -extrinsic pathways. Inhibiting hypoxia-inducible factor (HIF) function has recently been approved as a cancer treatment strategy. Hence, it is important to understand how regulators of HIF may affect tumor growth under physiological conditions. Here we report that in aging mice factor-inhibiting HIF (FIH), one of the most studied negative regulators of HIF, is a haploinsufficient suppressor of spontaneous B cell lymphomas, particular pulmonary B cell lymphomas. FIH deficiency alters immune composition in aged mice and creates a tumor-supportive immune environment demonstrated in syngeneic mouse tumor models. Mechanistically, FIH-defective myeloid cells acquire tumor-supportive properties in response to signals secreted by cancer cells or produced in the tumor microenvironment with enhanced arginase expression and cytokine-directed migration. Together, these data demonstrate that under physiological conditions, FIH plays a key role in maintaining immune homeostasis and can suppress tumorigenesis through a cell-extrinsic pathway.

## Significance

Under pathological conditions, hypoxia and the hypoxic tissue microenvironment influence cell behavior and resistance to therapies. Increasing numbers of modulators, including those targeting factor-inhibiting hypoxia-inducible factor [(HIF) (FIH)], an enzymatic inhibitor of HIF, have been developed to alter HIF activity and to treat diseases including cancer. Understanding how FIH functions under physiological conditions is therefore vital. Here, we identify FIH as a key regulator of immune homeostasis and suppressor of B cell lymphomagenesis throughout the physiological aging process. FIH deficiency in the host or in myeloid cells alone creates a tumor-supportive tumor microenvironment to promote cancer cell growth. Although the current work is limited to transgenic mouse studies, this information may inform future therapeutic application of HIF and FIH modulators.

The importance of hypoxia in influencing cancer cell behavior and cell fate has been well established. The hypoxic tumor microenvironment (TME) is one of the major causes of resistance to cancer therapies, in particular to radiotherapy (1). Additionally, the hypoxic TME can kill cancer cells by necrosis while also conferring several survival advantages that have been designated as hallmarks of cancer (2). Hence, there is intense interest in developing therapeutic strategies aimed at modulating tumor hypoxia. Mechanistically, cells respond to changes in oxygen levels by regulating the level and activity of the alpha subunits of hypoxia-inducible factor (HIF): HIF1α, HIF2α, and HIF3α. HIFα protein heterodimerizes with the beta subunit of HIF (HIF1β or ARNT) to control gene expression. The amount of HIFα protein is regulated by the HIF prolyl hydroxylase domain enzymes (PHDs), via post-translational hydroxylation of specific prolyl residues. Under normoxia, prolyl hydroxylation of HIFα enables association with the von Hippel–Lindau (VHL) E3 ligase complex, which results in its degradation via the ubiquitin–proteasome pathway. The importance of HIF signaling in cancer is evident by the frequent mutation of VHL in kidney cancers, leading to an increased level of HIF and related activity (3). Upregulation of HIF has also been associated with increased mortality in several other cancer types (4). Such findings have led to the development of various HIF inhibitors for cancer treatment (5, 6). Recently, Belzutifan (PT2977), a small molecule inhibitor that interferes with the heterodimerization of the HIF2α protein complex, was approved for treatment of renal cell carcinoma patients with germline VHL mutations (7). However, as HIF is also involved in many aspects of normal physiology, such as erythropoiesis, inhibition of HIF causes adverse effects such as anemia. A similar HIF2α inhibitor (PT2385) has been shown to impair ventilatory responses to hypoxia at therapeutic doses required for tumor inhibition in mice (8). To minimize the side effects of pan-HIF inhibition, selective modulation of the HIF pathway is emerging as an appealing approach.

Factor-inhibiting HIF (FIH) was originally identified as an interacting partner of HIF1α (9) and was subsequently found to inhibit the transcriptional activities of both HIF1α and HIF2α (10, 11). HIFα contains two transactivation domains, N-TAD and C-TAD, which are located at the N and C terminus of the proteins, respectively. Its C-TAD is asparaginyl-hydroxylated by the FIH enzyme under normoxia, which prevents its interaction with p300/CBP. FIH does not inhibit the transcription of all HIF target genes, rather it selectively inhibits HIF targets that are specifically regulated by C-TAD (12). The selective influence of FIH on HIF pathways is supported by the phenotype of mice with embryonic deletion of the FIH gene: FIH deficiency does not alter classical aspects of HIF function such as angiogenesis and erythropoiesis. Instead, it causes elevated metabolic rate, insulin sensitivity, and reduced body weight (13, 14). All these led to an emerging interest in targeting FIH to achieve selective modulation of HIF signaling (15). Thus, a deep understanding of the physiological importance of FIH, especially its role in tumorigenesis, is needed.

Varied effects of FIH on tumor development have been observed in different cancer cell lines. For example, FIH has been shown to be tumor-promoting by suppressing the p53/p21 axis, inhibiting HIF1α-dependent apoptosis, and enhancing angiogenesis (16–18). Conversely, FIH has been reported to inhibit cell proliferation and invasion in other cell lines, which might be linked with the activation of HIF1α (19–23). Most of these observations have not been confirmed in animal models and very little is known about the impact of FIH in non-malignant cells in the TME, immune cells and tumor stroma in particular. The TME affects many aspects of tumor development, and the HIF family of transcription factors are key regulators of many immune cell functions (24–26). Therefore, we hypothesized that FIH may influence tumorigenesis by regulating the functions of both cancer cells and their surrounding immune cells. Under normal physiological conditions, cancer is a disease associated with aging. Hence, it is crucial to investigate whether FIH plays a role in spontaneous tumorigenesis in vivo during the normal aging process. We report herein the generation and characterization of an FIH transgenic mouse cohort throughout the normal aging process. Necropsy analyses of aged FIH transgenic mice revealed spontaneous B cell lymphomas, suggestive of a tumor suppressive role of FIH. Analyses of syngeneic tumor growth in FIH-deficient mice support FIH’s role in regulating host immune homeostasis and the TME.

## Results

### FIH Is a Haploinsufficient Suppressor of B Cell Lymphomagenesis.

The biological importance of FIH under physiological conditions was examined in an aging cohort of FIH transgenic mice deficient of *Hif1an* gene exon 1 and 2 on a mixed C57BL/6; 129 background (*SI Appendix*, Fig. S1*A*). We also generated an anti-mouse FIH antibody using a truncated FIH protein that lacks disordered regions in its N terminus. The specificity of the antibody to mouse FIH was confirmed using tissue extracts derived from FIH wild-type (FIH^+/+^) and knockout (FIH^Δ1–2/Δ1–2^) mice. A specific protein band at ∼40 kDa, corresponding to the molecular weight of mouse FIH, was detected in various tissues derived from FIH^+/+^ but not FIH^Δ1-2/Δ1-2^ mice (*SI Appendix*, Fig. S1*B*). Similar levels of FIH were detected across all tissues tested from FIH^+/+^ animals (*SI Appendix*, Fig. S1 *B* and *C*).

**Fig. 1.**
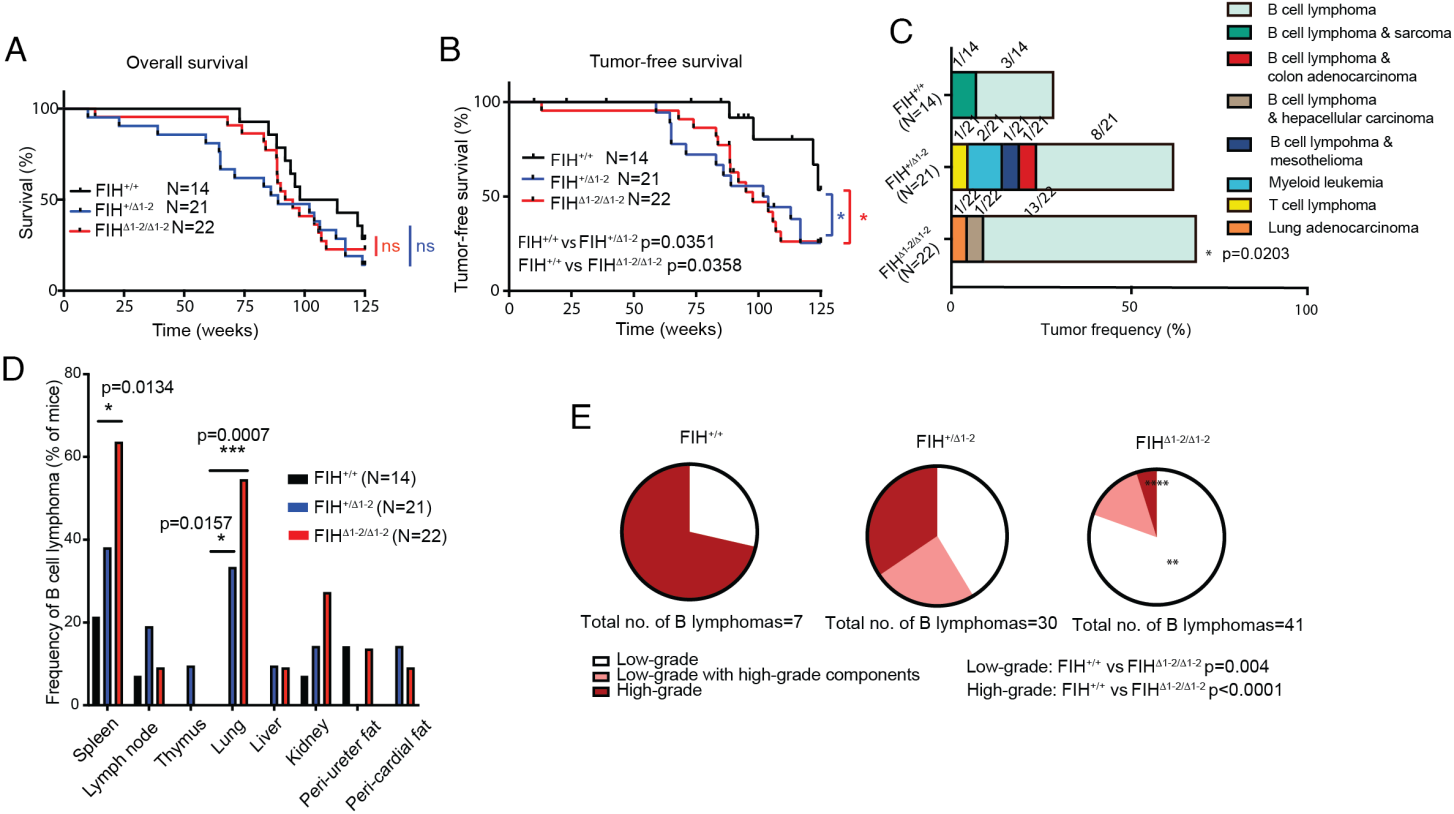
FIH deficiency increases spontaneous tumorigenesis. (*A*) Overall survival curve of FIH^+/+^ (N = 14) FIH^+/Δ1-2^ (N = 21), and FIH^Δ1-2/Δ1-2^ (N = 22) mice over 125 wk. ns, non-significant by the Mantel–Cox test. N denotes the number of mice. (*B*) Tumor-free survival curve of FIH^+/+^ (N = 14) FIH^+/Δ1-2^ (N = 21), and FIH^Δ1-2/Δ1-2^ (N = 22) mice over 125 wk. * indicates *P* < 0.05 by the Mantel–Cox test. Throughout the results, ns label and asterisks indicate the comparison between FIH^+/+^ and FIH^+/Δ1-2^ (blue) and between FIH^+/+^ and FIH^Δ1-2/^^Δ1-2^ (red), except where otherwise indicated. (*C*) The spectrum and frequency of tumors observed in FIH^+/+^ (N =14), FIH^+/Δ1-2^ (N = 21), and FIH^Δ1-2/Δ1-2^ (N = 22) animals. Eight cancer types are colored and tumor frequencies (number of mice with detectable tumors divided by number of mice with the indicated genotype) are indicated. FIH^Δ1-2/Δ1-2^ mice showed increased tumor incidence compared to FIH^+/+^ mice (*P = 0.0203, χ^2^ test). (*D*) B cell lymphoma frequency in indicated organs and tissues (number of mice with B cell lymphoma at the indicated site divided by number of mice with the indicated genotype) in FIH^+/+^(N = 14), FIH^+/Δ1-2^ (N = 21), and FIH^Δ1-2/Δ1-2^ (N = 22) animals (* indicates *P* < 0.05 and ** indicates *P* < 0.01 by the χ^2^ test). (*E*) Pie charts showing the proportions of B cell lymphomas of indicated grade (number of B cell lymphomas of indicated grade divided by the total number of B cell lymphomas found within the group) in FIH^+/+^ (n of B lymphomas=7), FIH^+/Δ1-2^ (n of B lymphomas=30) and FIH^Δ1-2/Δ1-2^ (n of B lymphomas = 41). ** indicates *P* < 0.01 and **** indicates *P* < 0.0001 compared with FIH^+/+^ by the χ^2^ test.

We monitored a cohort of FIH^+/+^ (n = 14), FIH^+/Δ1-2^ (n = 21) and FIH^Δ1-2/Δ1-2^ (n = 22) mice over a 125-wk period. While FIH expression status did not affect the lifespan of the experimental animals (Fig. 1*A*; upper case N denotes number of mice hereafter), necropsy analyses revealed that FIH^+/Δ1-2^ and FIH^Δ1-2/Δ1-2^ mice displayed accelerated tumor onset compared with FIH^+/+^ mice (FIH^+/+^ vs. FIH^+/Δ1-2^ *P* = 0.0351, FIH^+/+^ vs. FIH^Δ1-2/Δ1-2^ *P* = 0.0358 by the Mantel–Cox test) (Fig. 1*B*).

Histology and immunohistochemistry (IHC) analysis showed that B220^+^ B cell lymphomas accounted for over 87% of tumors examined, and occurred in all three genotypes: 4/14 FIH^+/+^, 10/21 FIH^+/Δ1-2^ and 14/22 FIH^Δ1-2/Δ1-2^ mice (Fig. 1*C* and *SI Appendix*, Fig. S1*D*). The incidence of B cell lymphomas was significantly higher in FIH^+/Δ1-2^ and FIH^Δ1-2/Δ1-2^ mice compared with FIH^+/+^ mice (Fig. 1*C*). A few B cell lymphoma-bearing FIH transgenic mice developed additional tumor types including follicular dendritic cell sarcoma (FDCS), colon adenocarcinoma, mesothelioma and hepatocellular carcinoma (Fig. 1*C*). We also observed myeloid leukemia (two FIH^+/Δ1-2^ mice), T cell lymphoma (one FIH^+/Δ1-2^ mouse) and lung adenocarcinoma (one FIH^Δ1-2/Δ1-2^ mouse) in the absence of B cell lymphoma.

The B cell lymphomas were detected in lymphoid and extra-nodal tissues such as the lung, liver, and kidney (*SI Appendix*, Fig. S1*E*). Among lymphoid tissues, the spleen was the most common site, with B cell lymphomas detected in 21.4 % (3/14) of FIH^+/+^, 38.1 % (8/21) of FIH^+/Δ1-2^ and 63.6 % (14/22) of FIH^Δ1-2/Δ1-2^ mice (Fig. 1*D*). Compared to the FIH^+/+^ animals, a significantly increased percentage of FIH^Δ1-2/Δ1-2^ was found to have splenic B cell lymphomas (*P* = 0.0134). Interestingly, pulmonary B cell lymphomas were detected at a high frequency in FIH^+/Δ1-2^ (7/21; 33.3%) and FIH^Δ1-2/Δ1-2^ (12/22; 54.6%) mice, with none in FIH^+/+^ mice (0/14; 0%). All mice with pulmonary B cell lymphoma exhibited lymphoma in at least one other tissue (*SI Appendix*, Table S1), reflecting the circulatory nature of hematological malignancies.

Histologically, B cell lymphomas are categorized into three groups (exemplars shown in *SI Appendix*, Fig. S1*F*): 1) low-grade B cell lymphomas are associated with good patient outcomes (27) and consist of small, mildly pleomorphic cells (black arrows); 2) high-grade lymphomas are the most aggressive type which shorten life span and are large with prominent nuclei (white arrows); and 3) mixed low- and high-grade B cell lymphomas share features of both. Based on these histological criteria, 5/7 B cell lymphomas derived from FIH^+/+^ mice were high-grade B cell lymphoma; FIH^+/Δ1-2^ mice showed mixed grades; and FIH^Δ1-2/Δ1-2^ mice showed a majority (33/41) of low-grade B cell lymphomas (Fig. 1*E* and *SI Appendix*, Fig. S1*G*). These data suggest that under normal physiological conditions, FIH has an inhibitory effect on spontaneous B cell lymphomagenesis in aging mice. The findings that FIH^+/Δ1-2^ and FIH^Δ1-2/Δ1-2^ mice developed B cell lymphomas with similar tumor onset and spectrum (Fig. 1 *B*–*D*) suggest that FIH may be a haploinsufficient tumor suppressor in aging mice.

### FIH Deficiency Alters Immune Cell Composition in Aged Mice.

Development of B cell lymphomas can be caused by intrinsic defects in B cells or extrinsic dysfunction of the tissue microenvironment, or a combination of both. As FIH^+/Δ1-2^ mice already showed increased incidence of B cell lymphomas, we examined B cell functions by comparing proliferation and survival potential of splenic B cells derived from FIH^+/+^ and FIH^+/Δ1-2^ mice in vitro. Various stimuli were used: anti-CD40 monoclonal antibody to activate B cell signaling; lipopolysaccharide (LPS) to mimic bacterial infection; and IgM antibodies, which elicit aggregation of the B cell receptor and mimic the initial signal to activate B cells. Although FIH^+/Δ1-2^ B cells showed a very small reduction in proportion of apoptotic cells at baseline (2.6% vs. 2.0%, *P* = 0.036), their propensity for apoptosis (Annexin V^+^, Viability dye^−^) and cell death (Annexin V^int^, Viability dye^+^) in response to stimuli was very similar to FIH^+/+^ derived B cells (*SI Appendix*, Fig. S2*A*). FIH heterozygosity also had minimal impact on proliferation of splenic B cell subtypes, namely the follicular B cells and marginal zone B cells, as determined by flow cytometric analysis of cell division by dilution of carboxyfluorescein diacetate succinimidyl ester (CFSE) (*SI Appendix*, Fig. S2 *B* and *C*). This suggests that FIH haploinsufficiency does not cause profound B cell dysfunction that drives B cell lymphomagenesis.

**Fig. 2.**
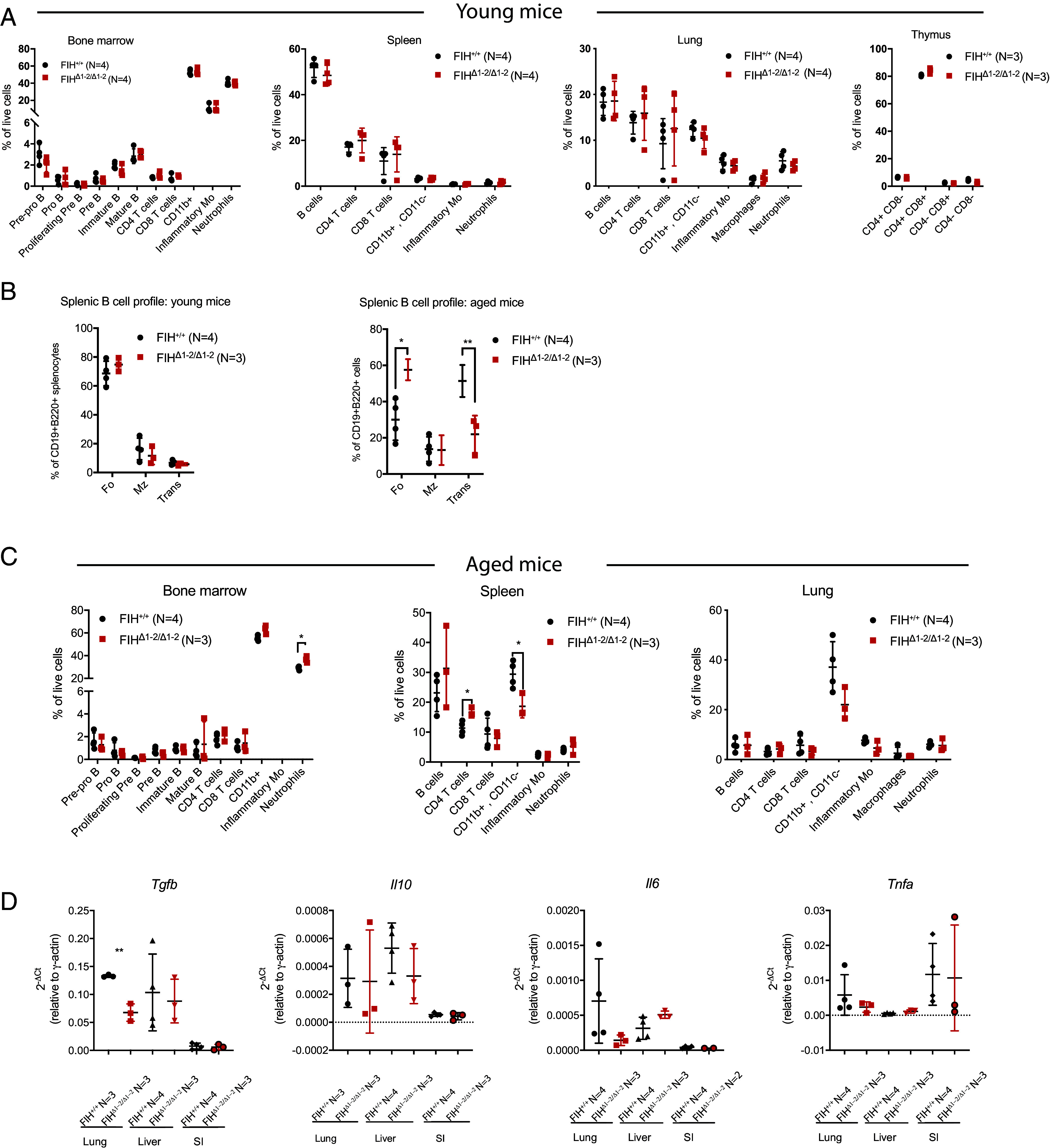
FIH deficiency affects the immune cell composition in aged mice but not young adult mice. (*A*) Flow cytometry profiling of immune cells (pre-pro B cells (B220^+^CD43^+^CD24^lo^BP-1^−^IgM^−^IgD^−^), pro B cells (B220^+^CD43^+^CD24^mid^BP-1^−^IgM^−^IgD^−^), proliferating pre B cells (B220^+^CD43^+^CD24^hi^BP-1^+^IgM^−^IgD^−^), pre B cells (B220^+^CD43^−^CD24^hi^BP-1^+^IgM^−^IgD^−^), immature B cells (B220^+^CD43^−^CD24^mid^IgM^+^IgD^−^), mature B cells (B220^+^CD43^−^CD24^lo^IgM^+^IgD^+^), CD4^+^ T cells, CD8^+^ T cells, CD11b^+^ myeloid cells, inflammatory monocytes (CD11b^+^Ly6G^−^Ly6C^hi^), and neutrophils (CD11b^+^Ly6G^+^Ly6C^+^) in the indicated tissues of 18-wk-old (young) FIH^+/+^ and FIH^Δ1-2/Δ1-2^ mice (Bone marrow, spleen, and lung tissues were derived from 4 FIH^+/+^ mice and 4 FIH^Δ1-2/Δ1-2^ mice, whereas thymus tissues were obtained from 3 FIH^+/+^ and 3 FIH^Δ1-2/Δ1-2^ mice). (*B*) Flow cytometry analysis of B cell subsets in the spleens of 18-wk-old (young, *Left*) and 104-wk-old (aged, *Right*) mice FIH^+/+^ mice (N = 4) and FIH^Δ1-2/Δ1-2^ mice (N = 3). Follicular (Fo) B cells (CD19^+^B220^+^CD21^mid^CD23^+^), marginal zone (Mz) B cells (CD19^+^B220^+^CD21^+^CD23^mid^), transitional (Trans) B cells (CD19^+^B220^+^CD21^−^CD23^−^). Inflammatory Mo, inflammatory monocytes. (*C*) Flow cytometry profiling of immune cell subtypes in the indicated tissues of 104-wk-old (aged) FIH^+/+^ (N = 4) and FIH^Δ1-2/Δ1-2^ (N = 3) mice. (*D*) mRNA levels of *Tgfb*, *Il10, Il6,* and *Tnfa* in the lung, liver, and small intestine (SI) of FIH^+/+^ and FIH^Δ1-2/Δ1-2^ mice of 104 wk (aged) determined by RT-qPCR. Horizontal lines indicate mean with SD. * indicates *P* < 0.05, ** indicates *P* < 0.01 by two-tailed *t* tests. Outliers identified by the Grubbs test were excluded from the dataset.

Aging is accompanied by immune senescence, which is closely related to development of malignant tumors (28). As B cell lymphomas predominantly occur in aged FIH-deficient mice, we hypothesized that FIH deficiency may disturb immune balance more in aged mice than in young mice. We profiled the immune cells of young (18-wk-old) and aged (104-wk-old) FIH^+/+^ and FIH^Δ1-2/Δ1-2^ mice using flow cytometry. Comparing FIH^+/+^ with FIH^Δ1-2/Δ1-2^ mice of 18 wk old, we observed very few differences in the numbers of various types of B cells (including pre-pro B, pro B, proliferating pre-B, pre-B, immature B, and mature B cells), T cells and myeloid cells examined in bone marrow, spleen, thymus and lung tissues (Fig. 2*A*). Splenic B cell profiling in young mice also did not show differences in the levels of various B cell subtypes (follicular B cells, marginal zone B cells, transitional B cells; Fig. 2 *B*, *Left* and *SI Appendix*, Fig. S2*D*), suggesting that FIH may not play an essential role in regulating B cell differentiation in young mice in vivo.

Intriguingly, in the spleen of aged FIH^Δ1-2/Δ1-2^ mice, the proportions of various B cell types were altered in comparison to those detected in FIH^+/+^ mice. Follicular B cells (FIH^+/+^ 30% and FIH^Δ1-2/Δ1-2^ 57.6%, *P* = 0.0129) were increased in aged FIH^Δ1-2/Δ1-2^ mice, whereas transitional B cells (FIH^+/+^ 51.4% and FIH^Δ1-2/Δ1-2^ 21.97%, *P* = 0.0096) were decreased (Fig. 2 *B*, *Right* and *SI Appendix*, Fig. S2*D*). Aged FIH^Δ1-2/Δ1-2^ mice also exhibited elevated percentages of bone marrow neutrophils (Fig. 2*C* and *SI Appendix*, Fig. S2*E*; FIH^+/+^ 29% and FIH^Δ1-2/Δ1-2^ 36.6%, *P* = 0.0058) and splenic CD4 T cells (FIH^+/+^ 11.4% and FIH^Δ1-2/Δ1-2^ 16.6%, *P* = 0.0196) and reduced splenic CD11b^+^ CD11c^−^ myeloid cells (FIH^+/+^ 29.4% and FIH^Δ1-2/Δ1-2^ 18.6%, *P* = 0.0204). Although we found reduced CD11b^+^ CD11c^-^ myeloid cells in lung tissues of aged FIH^Δ1-2/Δ1-2^ mice compared to FIH^+/+^ mice, it was not statistically significant. These results suggest that FIH deficiency alters immune cell composition, mainly in aged mice.

To further characterize the immune status of the aged animals, we examined cytokine levels in organs vulnerable to external stimuli (lung, liver, and small intestine). Notably only lung, not liver or small intestine tissues of FIH^Δ1-2/Δ1-2^ mice showed significantly reduced expression of *Tgfb* (Fig. 2*D*; FIH^+/+^ 0.1329 and FIH^Δ1-2/Δ1-2^ 0.06753, *P* = 0.01976), a cytokine that has been shown to induce senescence of B cell lymphoma cells (29), suggesting that FIH deficiency may generate an immune environment that favors B cell growth in the lung, consistent with pulmonary B cell lymphomas being only detected in FIH-defective mice.

### Reduced FIH Gene Dosage in the Host Enhances Tumorigenesis in Syngeneic Tumor Models.

To test the hypothesis that FIH may suppress tumorigenesis by altering the TME via a cell-extrinsic pathway, we used C57BL6 FIH transgenic mouse cohorts and adopted a subcutaneous syngeneic murine tumor model. Lewis lung carcinoma (LLC) cells have C57BL6 genetic background and are known to cause immune cell infiltration upon inoculation (30). LLC cells were injected subcutaneously into a cohort of FIH^+/+^, FIH^+/Δ1-2^, and FIH^Δ1-2/Δ1-2^ mice of 8 to 14 wk (full data shown in *SI Appendix*, Table S2). As young FIH mice did not exhibit detectable impacts on immune cell composition (Fig. 2 *A* and *B*), we anticipate that any observed differences in tumor growth in this syngeneic tumor model would reflect the potential of FIH in regulating immune homeostasis and TME. As expected, LLC tumors grown in FIH^+/+^ mice have foci of infiltrating inflammatory cells including F4/80-positive macrophages, FOXP3-positive regulatory T cells (Treg), CD4 and CD8 T cells (an example of LLC tumor grown in FIH^+/+^ is shown in *SI Appendix*, Fig. S3*A*), confirming that the syngeneic LLC subcutaneous tumor model is suitable for studying the role of FIH on TME and tumorigenesis. Many infiltrating macrophages are positive for M2-macrophage marker arginase (ARG1) but negative for M1-macrophage marker nitric oxide synthase 2 (NOS2), indicative of a tumor-supportive microenvironment.

**Fig. 3.**
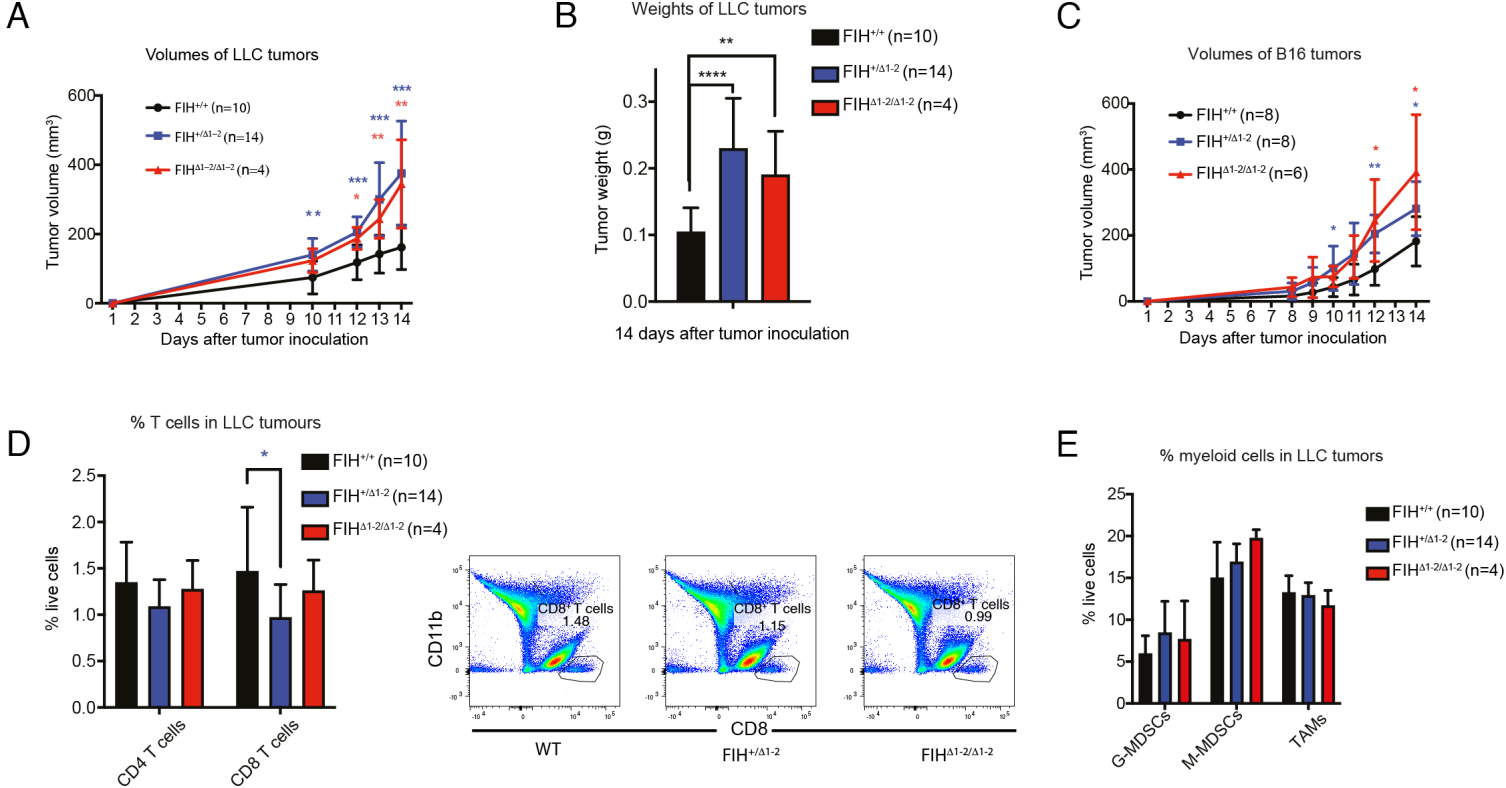
FIH-defective mice show enhanced growth of LLC tumors. FIH^+/+^, FIH^+/Δ1-2^, and FIH^Δ1-2/Δ1-2^ mice were subjected to subcutaneous injection of 4 × 10^5^ LLC cells or 1 × 10^5^ B16 cells on both flanks. n denotes number of tumors. (*A*) Average LLC tumor volumes from day 1 to day 14. Tumor volumes and number of tumors measured at each time point are shown in *SI Appendix*, Table S2. Statistically significant differences between FIH^+/+^ vs. FIH^+/Δ1-2^ and FIH^Δ1-2/Δ1-2^ are indicated by blue and red asterisks, respectively. (*B*) Average weights of LLC tumors collected on day 14. Tumor weights are shown in *SI Appendix*, Table S2. (*C*) Mean B16 tumor volumes from day 1 to 14 are shown. Tumor volumes, number of tumors measured and number of mice with measurable tumors at each time point are shown in *SI Appendix*, Table S3. (*D*) Flow cytometry analysis of the percentages of CD4 and CD8 T cells in all LLC tumors collected on day 14 post inoculation (*left*). Representative FACS plots of CD8 T cells (*right*). (*E*) Flow cytometry analysis of the percentages of tumor-associated macrophages (TAMs, CD11b^+^F4/80^+^), monocytic-myeloid derived suppressor cells (M-MDSCs, CD11b^+^CD11c^−^Ly-6G^−^Ly6C^hi^), and granulocytic-myeloid derived suppressor cells (G-MDSCs, CD11b^+^CD11c^−^Ly-6G^+^Ly6C^+^) in LLC tumors collected on day 14. Bars indicate mean with SD. * indicates *P* < 0.05, by two-tailed *t* tests.

Compared to the FIH^+/+^ group, LLC tumors grown in FIH^+/Δ1-2^ and FIH^Δ1-2/Δ1-2^ mice had greater volume from 10 d onward (Fig. 3*A* and *SI Appendix*, Table S2). LLC tumors from FIH^+/Δ1-2^ and FIH^Δ1-2/Δ1-2^ mice also had increased weights when resected at the end of study compared with those from FIH^+/+^ mice (Fig. 3*B*: FIH^+/+^ vs. FIH^+/Δ1-2^ 0.10 g vs. 0.23 g, *P* < 0.0001, FIH^+/+^ vs. FIH^Δ1-2/Δ1-2^ 0.10 g vs. 0.19 g, *P* = 0.007; *t* test; lower case n denotes number of tumors hereafter).

To explore this result in another tumor model, we injected B16 melanoma cells into another cohort of FIH^+/+^, FIH^+/Δ1-2^, and FIH^Δ1-2/Δ1-2^ mice (mean tumor sizes at each time point shown in *SI Appendix*, Table S3). Like LLC tumors, B16 tumors grown in FIH^+/Δ1-2^ and FIH^Δ1-2/Δ1-2^ mice showed increased volumes at multiple time points compared to the FIH^+/+^ group (Fig. 3*C* and *SI Appendix*, Table S3). Unlike LLC tumors, B16 tumors were liquid in consistency and highly necrotic, with extensive areas of anucleate and eosinophilic cells on histological examination (*SI Appendix*, Fig. S3*B*), preventing accurate measurement of tumor weight. Nevertheless, the data from these two tumor types indicate that reduced FIH gene dosage in the host promotes tumor growth in a non-cell autonomous manner.

To investigate the possibility that FIH deficiency in the host influences the tumor immune environment, we used flow cytometry to measure the tumor-infiltrating immune cells in all LLC tumors collected from all three host genotypes at 14 d post injection. The percentage of CD8 T cells was significantly lower in LLC tumors harvested from FIH^+/Δ1-2^ than from FIH^+/+^ mice (1.47% vs. 0.97% respectively, *P* = 0.03, *t* test), whereas the percentage of CD4 T cells was similar in all tumors grown in all three FIH genotype groups (Fig. 3*D*). FIH status, however, had minimal impact on the percentage of tumor-associated myeloid subtypes present in LLC tumors, granulocytic-myeloid derived suppressor cells (G-MDSC, CD11b^+^ Ly-6G^+^ Ly-6C^int^), monocytic MDSCs (M-MDSC, CD11b^+^ Ly-6G^−^ Ly-6C^hi^), or tumor-associated macrophages (TAMs, CD11b^+^ F4/80^+^) (Fig. 3*E*). Notably, adding all CD11+ myeloid cells together accounts for up to 40% of live cells detected in LLC tumors (Fig. 3*E*). Given the evidence for a potential effect of FIH on the immune environment, despite apparently normal numbers of myeloid cells, we hypothesized that there might be an FIH-dependent functional abnormality in these cells.

### FIH-Defective Myeloid Cells in the Host Can Create a Tumor Supporting Immune Microenvironment.

**Fig. 4.**
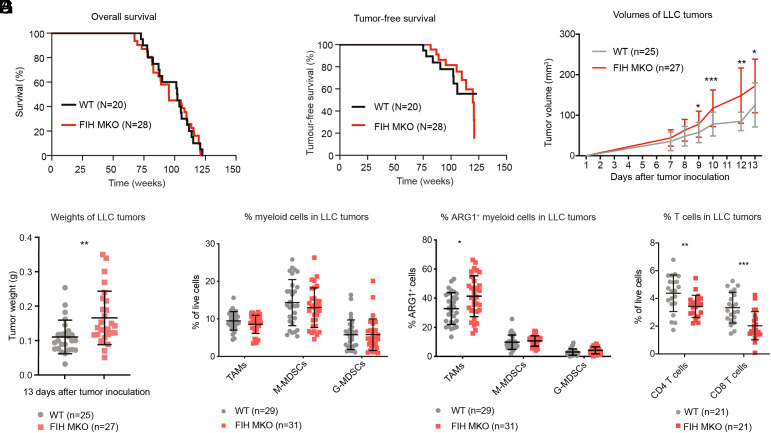
FIH myeloid knockout (MKO) mice show enhanced subcutaneous growth of LLC tumors but not spontaneous tumor development. (*A* and *B*) A cohort of WT (N = 20) and FIH MKO (N = 28) mice were monitored for 125 wk. The overall survival (*A*) and tumor-free survival curves (*B*) are shown. (*C*–*G*) WT and FIH MKO mice were subjected to subcutaneous injection of 4 × 10^5^ LLC cells at the left flank. (*C*) Average tumor volumes from day 1 to 13. Mean tumor sizes at each time point are shown in *SI Appendix*, Table S4. (*D*) Average weights of LLC tumors from WT and FIH MKO animals at the end of the study (pool of four studies). Individual points represent single tumors. (*E*) Quantification of TAMs, M-MDSCs, and G-MDSCs in LLC tumors by flow cytometry (pool of four studies). (*F*) Average percentage of TAMs, M-MDSCs, and G-MDSCs in LLC tumors positive for arginase 1 (ARG1) determined by flow cytometry (pool of four studies). (*G*) Average percentage of CD4 and CD8 T cells from LLC tumors measured by flow cytometry (pool of three studies). Upper case N denotes number of animals while lower case n denotes number of tumors hereafter. Error bars indicate the mean ± SD in panels *C*–*G*. * indicates *P* < 0.05, ** indicates *P* < 0.01, and *** indicates *P* < 0.001, two-tailed *t* test.

Given that myeloid cells are key players in the TME through their ability to either promote or suppress tumor growth (31) and are the most abundant type of tumor-infiltrating cells in our mouse models (Fig. 3*E*), we explored the potential role of myeloid cell FIH in regulating the TME. We generated myeloid-specific FIH knockout mice, denoted as FIH MKO to distinguish from global FIH deletion (FIH^Δ1-2/Δ1-2^). Bone marrow–derived macrophages (BMDMs) isolated from FIH MKO showed an approximate 50% reduction in FIH expression (*SI Appendix*, Fig. S4*A*), with more pronounced FIH reduction occurring in bone marrow neutrophils (*SI Appendix*, Fig. S4*B*). A cohort of WT FIH (N = 20) and FIH MKO (N = 28) mice were observed over 125 wk for spontaneous tumorigenesis. Reduced FIH expression in the myeloid compartment alone did not affect the lifespan (Fig. 4*A*), tumor onset (Fig. 4*B*) or location of the tumors (*SI Appendix*, Fig. S4 *C* and *D*) in FIH MKO mice compared to WT mice.

In aged animals (104 to 113 wk old), similar percentages of lymphocytic and myeloid subtypes were observed in the spleen and liver (*SI Appendix*, Fig. S4 *E* and *F*) of WT and FIH MKO mice, consistent with similar tumor incidence in these locations (*SI Appendix*, Fig. S4 *C* and *D*). These results suggest that partial deletion of FIH in myeloid cells alone may not be sufficient to predispose animals to spontaneous tumorigenesis under physiological conditions. Nonetheless, we cannot rule out that a complete ablation of FIH in myeloid cells might result in a tumorigenic phenotype.

We next used the LLC syngeneic tumor model to explore whether the tumor-supportive immune environment seen in FIH^Δ1-2/Δ1-2^ mice can also be recapitulated in FIH MKO mice. LLC tumor cells were subcutaneously injected to the single flank of FIH WT (N = 25) and MKO (N = 27) mice across four independent experiments. Reduced FIH expression in F4/80^+^ TAMs was confirmed by western blotting of TAMs isolated by magnetic-activated cell sorting (MACS) from LLC tumors in WT and FIH MKO mice (both N = 2, *SI Appendix*, Fig. S4*G*). The tumors in the FIH MKO animals had a significantly larger volume (FIH MKO 78.58 mm^3^ vs. WT 57.3 mm^3^, *P* = 0.012) than those in WT mice from day 9 onward (Fig. 4*C* and *SI Appendix*, Table S4). Similarly, the average weight of all tumors derived at the end of the study from the FIH MKO mice was greater than from WT mice (WT 0.1105 g, FIH MKO 0.1659 g, *P* = 0.0037; *t* test; Fig. 4*D*). Increased tumor volumes were also observed in FIH MKO mice inoculated with B16 tumors (WT vs. FIH MKO: 202.4 mm^3^ vs. 337.1 mm^3^, *P* = 0.03; *t* test) (*SI Appendix*, Fig. S4*H* and Table S5). This observation in FIH MKO is consistent with that seen in germline-deleted, globally FIH-deficient mice (Fig. 3 *A* and *C*), demonstrating the importance of myeloid FIH expression in suppressing tumor growth.

To further explore the influence of tumor-infiltrating immune cells on tumor growth, we examined the immune cell profiles of LLC tumors by FACS. Again, similar abundances of TAMs, M-MDSCs, and G-MDSCs were detected in LLC tumors derived from WT (n = 29) and FIH MKO (n = 31) (Fig. 4*E*), suggesting that myeloid expression of FIH may suppress tumor growth by influencing myeloid cell function rather than differentiation or proliferation.

Arginase 1 (ARG1), an enzyme that converts l-arginine to urea and l-ornithine, is highly expressed in TAMs and MDSCs. ARG1 has been shown to inhibit T cell proliferation by depleting l-arginine, an essential amino acid for T cell function, in the tumor microenvironment (32). Hence, enhanced ARG1 expression often associates with suppressive immune microenvironments. We therefore used a specific antibody to detect ARG1-expressing TAMs, M-MDSC and G-MDSCs derived from LLC tumors grown in the WT vs. FIH MKO mice. Notably, an increased percentage of ARG1-expressing TAMs was found in LLC tumors grown in FIH MKO mice compared to WT mice (TAM: WT vs. FIH MKO 32.9% vs. 41.3%, *P* = 0.0116, *t* test; Fig. 4*F*). These findings suggest that although partial deletion of FIH did not affect the number of tumor-infiltrating TAMs, it might alter the function of TAMs. Consistent with the notion that ARG1-expressing TAMs suppress T cell proliferation (32), the LLC tumors grown in FIH MKO mice (n = 21) had a small but detectable reduction in the proportion of both CD4 and CD8 T cells than tumors in WT mice (n = 21) (CD4 T cells: WT 4.38%, FIH MKO 3.43%, *P* = 0.0076; CD8 T cells: WT 3.34%, FIH MKO 2.03%, *P* = 0.00028; *t* test) (Fig. 4*G*). Together, these data illustrate that myeloid-specific deletion of FIH, although incomplete and insufficient to initiate spontaneous tumor growth, can still provide a tumor-promoting microenvironment.

### FIH and HIF2α Interaction in Myeloid Cells Influences the Tumor Microenvironment.

One of the best-known functions of FIH is to inhibit the transcriptional activities of HIF. As the involvement of HIF1α and HIF2α in tumorigenesis differs (33), we investigated the impact of myeloid-expressed HIF1α or HIF2α on immune compositions in aged mice with myeloid-specific knock-outs of HIF1α or HIF2α gene in vivo. In the spleen and liver of aged (>88 wk) animals, deletion of HIF1α in myeloid cells had little impact on the lymphocyte and myeloid cell compositions (*SI Appendix*, Fig. S5 *A* and *B*). In the spleen of HIF2α MKO mice, we observed an increase in the percentage of γδT cells, whereas other immune cell types were similar between HIF2α MKO and WT mice (*SI Appendix*, Fig. S5*C*). The immune cell compositions in the liver of WT and HIF2α MKO mice were also similar (*SI Appendix*, Fig. S5*D*).

**Fig. 5.**
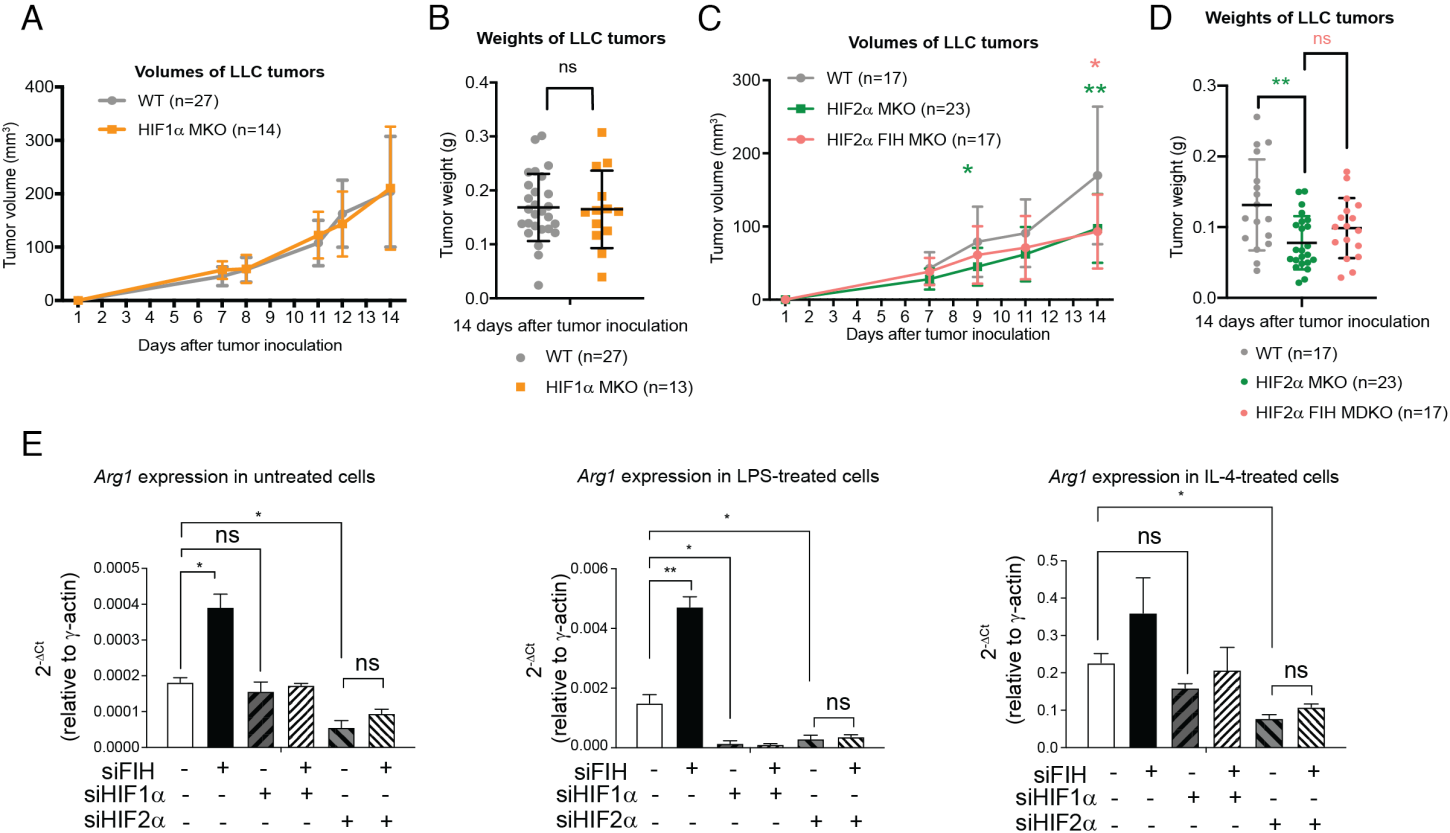
Myeloid deletion of HIF2α suppresses LLC tumor growth and abolishes the effect of myeloid FIH expression. (*A* and *B*) 4 × 10^5^ LLC cells were administered to the left flank of WT (N = 27) and HIF1α MKO (N = 14) animals (9 to 13 wk old). Pooled results from two independent experiments are presented. (*A*) Average tumor volumes from day 1 to 14. Mean tumor volumes at each time point are detailed in *SI Appendix*, Table S6. (*B*) Average weights of LLC tumors from WT and HIF1α MKO animals at the end of the study. (*C* and *D*) 4 × 10^5^ LLC cells were administered to the left flank of WT (N = 17), HIF2α MKO (N = 23), and HIF2α FIH MDKO (myeloid double knockout) (N = 17) animals (9 to 13 wk old). Pooled results from three independent experiments are presented. (*C*) Average tumor volumes from day 1 to 14. Mean tumor volumes at each time point are shown in *SI Appendix*, Table S7. * indicates *P* < 0.05 and ** indicates *P* < 0.01 by the two-tailed *t* test. Asterisks denote comparison between WT and HIF2α MKO mice (green) or between WT and HIF2α FIH MDKO mice (orange). (*D*) Average weights of LLC tumors from WT, HIF2α MKO and HIF2α FIH MDKO animals at the end of the study. Mean ± SD is indicated in all panels. ** indicates *P* < 0.005 by the two-tailed *t* test. (*E*) Murine macrophage cell line J774 cells were knocked down with either FIH, HIF1α, or HIF2α, or a combination of FIH/HIF1α or FIH/HIF2α, followed by stimulation with LPS or IL-4 for 48 h. *Arg1* expression was detected by qRT-PCR.

The ability of HIF1α or HIF2α deficient myeloid cells to influence tumor growth was tested using the subcutaneous LLC syngeneic mouse model. Compared to WT mice (n = 27), similar tumor volume and tumor weight were observed in HIF1α MKO mice (n = 14) (Fig. 5 *A* and *B* and *SI Appendix*, Table S6). In contrast, reduced tumor volume and tumor weight were observed in HIF2α MKO mice (n = 23) compared to that seen in WT mice (n = 17). In mice with no myeloid HIF2α expression, concomitant depletion of myeloid FIH has no impact on tumor volume and weight (Fig. 5 *C* and *D* and *SI Appendix*, Table S7), suggesting that the previously noted tumor suppressive effect of FIH could be mediated by HIF2α.

The transcriptional activities of both HIF1α and HIF2α are inhibited by FIH, and HIF1α/FIH and HIF2α/FIH will regulate the expression of three groups of target genes: HIF1α/FIH-specific, HIF2α/FIH-specific, and common target genes. To provide molecular evidence that HIF2α/FIH plays a more prominent role than HIF1α/FIH at regulating macrophage function, we used a murine macrophage cell line J774 in combination with RNAi of FIH, HIF1α, or HIF2α alone or in combination, as indicated. ARG1 expression was induced in J774 cells when cultured in IL-4 containing medium and both HIF1α-RNAi or HIF2α-RNAi reduced ARG1 expression (*SI Appendix*, Fig. S5*E*). Additionally, HIF1α-RNAi reduces *Arg1* expression only in J774 cells cultured in LPS medium, whereas HIF2α RNAi reduces *Arg1* expression in J774 cells cultured in all conditions tested (*SI Appendix*, Fig. S5*F* and Fig. 5*E*). Under the same conditions, RNAis of HIF1α or HIF2α have very small impact on the expression of *Vegf*, a known HIF target. FIH RNAi-induced *Arg1* expression can be reversed by either HIF1α RNAi or HIF2α RNAi, however, the effect of HIF2α-RNAi is more pronounced (Fig. 5*E*). All these are consistent with the notion that HIF1α and HIF2α may have differing influences on the regulation of gene expression profiles in macrophages in response to various stimuli, and that FIH/HIF2α may play a more prominent role than FIH/HIF1α in regulating *Arg1* expression in tumor promoting macrophages.

### FIH Inhibits *Arg1* Expression in Macrophages Treated with LPS and LLC-Conditioned Medium.

**Fig. 6.**
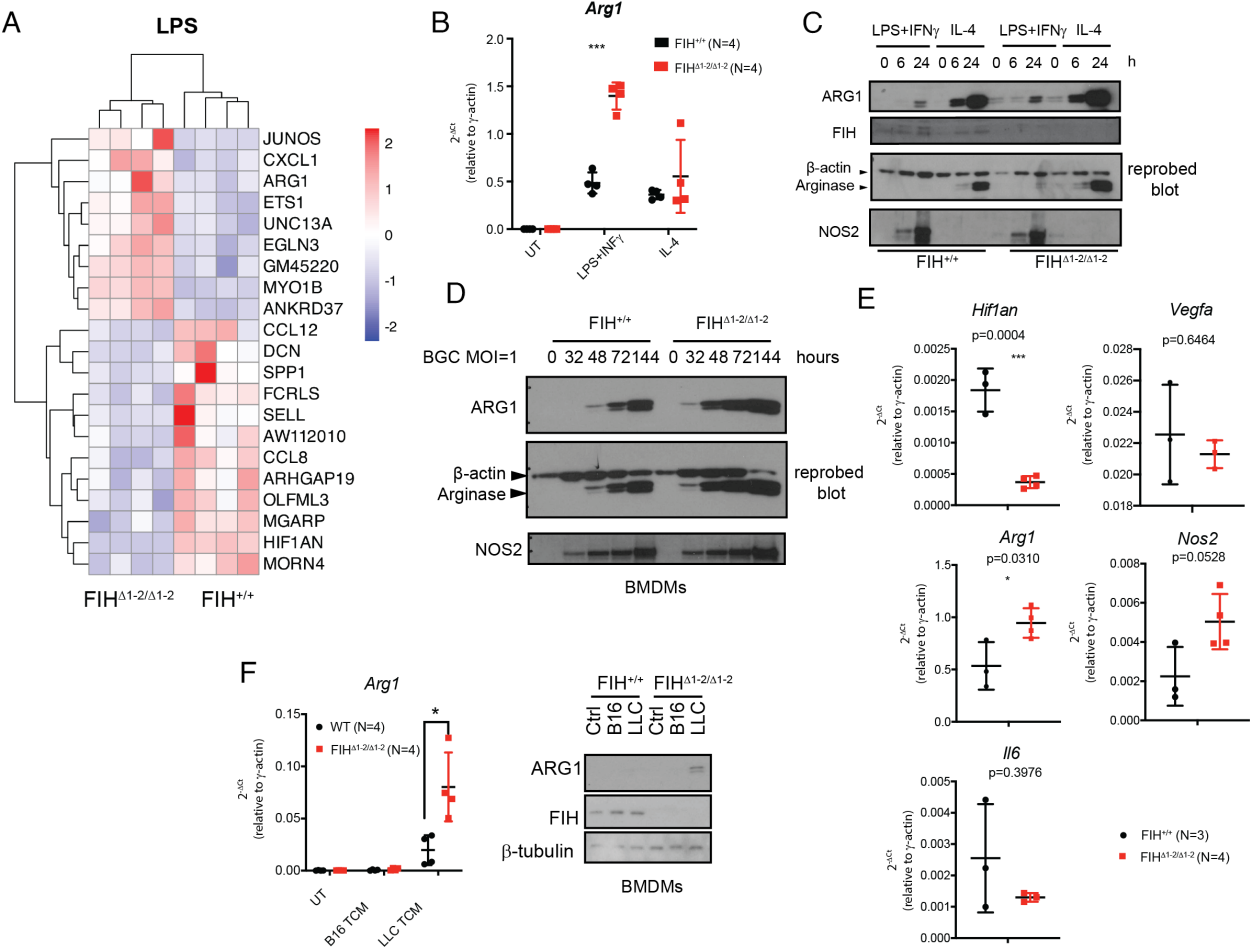
FIH deletion enhances *Arg1* expression in vitro. (*A*) BMDMs harvested from FIH^+/+^ (N = 4) and FIH^Δ1-2/Δ1-2^ (N = 4) mice were stimulated with LPS followed by bulk RNA sequencing. A clustered heat map of the differentially expressed genes from the FIH^+/+^ and FIH^Δ1-2/Δ1-2^ mice is shown. A gene was considered to be differentially expressed if its absolute log2 fold change is >1 and false discovery rate (FDR)< 0.05. Color key indicates the *z*-scores of normalized expression values. (*B*) BMDMs isolated from FIH^+/+^ and FIH^Δ1-2/Δ1-2^ mice were treated with M1–polarizing (5 ng/mL LPS and 1 ng/mL IFNγ) or M2–polarizing (10 ng/mL IL-4) agent for 24 h. mRNA level of *Arg1* was determined by RT-qPCR. UT, untreated. Bars indicate mean ± SD. **** indicates *P* < 0.0001 by two-tailed *t* tests. (*C*) Western blots showing ARG1 and NOS2 protein levels in FIH^+/+^ and FIH^Δ1-2/Δ1-2^ BMDMs in response to LPS+IFN or IL-4 treatments at the indicated time points. Following the detection of ARG1 expression, the same blot was incubated with anti-β-actin antibody as a loading control. (*D*) Western blots showing the expression levels of ARG1 and NOS2 in FIH^+/+^ and FIH^Δ1-2/Δ1-2^ BMDMs infected with *Mycobacterium bovis* BCG at one multiplicity of infection (MOI) and collected at indicated hours post infection. β-actin was used as a loading control in the re-probed blot. (*E*) RT-qPCR analysis of *Hif1an*, *Vegf*, *Arg1*, *Nos2*, and *Il6* expression in TAMs derived from LLC tumors grown in FIH^+/+^ (N = 3) and FIH^Δ1-2/Δ1-2^ (N = 4) mice. (*F*) FIH^+/+^ and FIH^Δ1-2/Δ1-2^ BMDMs were treated with medium conditioned by LLC or B16 cells (tumor-conditioned medium, TCM). *Arg1* expression was detected by RT-qPCR (*Left*) and western blotting (*Right*, β-tubulin as loading control). Horizontal lines and error bars represent mean with SD. * indicates *P* < 0.05 by two-tailed *t* tests.

To understand the mechanisms by which reduced FIH activity influences myeloid cell function to facilitate tumor growth, we compared mRNA profiles in bone marrow–derived macrophages (BMDMs) from FIH^+/+^ and FIH^Δ1-2/Δ1-2^ mice by a shallow RNA-sequencing. A small number of prominent differentially expressed transcripts were detected in FIH^+/+^ and FIH^Δ1-2/Δ1-2^ BMDMs (*SI Appendix*, Fig. S6*A*). LPS was used in BMDMs to induce polarization of macrophages toward a proinflammatory M1 state. The gene expression profiles of FIH^+/+^ and FIH^Δ1-2/Δ1-2^ BMDMs differed substantially more when the cells were treated with LPS compared to untreated (Fig. 6*A* vs. *SI Appendix*, Fig. S6*A*). Among the top differentially expressed transcripts in LPS-treated FIH^Δ1-2/Δ1-2^ BMDMs, two of the most well-known HIF targets, ANKRD37 and EGLN3 (15), were up-regulated, consistent with FIH being a transcriptional inhibitor of HIF. Interestingly, mRNAs of CXCL1, a chemokine known to recruit neutrophils, and ARG1 were up-regulated in FIH^Δ1-2/Δ1-2^ cells after LPS treatment compared with FIH^+/+^ (Fig. 6*A*). Some other differentially expressed genes are less well characterized, such as JUNOS (antisense Jun, lincRNA), UNC13A (neural transmitter and vesical maturation during exocytosis), GM45220 (unclassified gene), and Myo1b (unconventional myosin 1b).

The finding that *Arg1* was one of the top 9 up-regulated genes induced by LPS in FIH^Δ1-2/Δ1-2^ BMDMs is important, since elevated ARG1 is often used as a marker of tumor promoting macrophages (34). This also agrees with our finding in Fig. 5 that HIF/FIH regulates *Arg1* expression in J774 cells. The most well-known pathway for *Arg1* induction is through IL-4-STAzzT6, an anti-inflammatory and tumor-supportive signaling pathway (35). LPS+IFNγ can also induce *Arg1* via the TLR-MyD88 pathway during mycobacteria infection (36). We thus examined more closely the impact of FIH on *Arg1* expression in BMDMs. Both LPS+IFNγ and IL-4 induced *Arg1* expression (Fig. 6 *B* and *C*). However, only the induction of *Arg1* by LPS+INFγ, but not IL-4, was further enhanced by the absence of FIH at both mRNA and protein levels (Fig. 6 *B* and *C*). In contrast to ARG1, NOS2 in macrophages is pro-inflammatory and tumor suppressive (37). FIH status has minimal impact on NOS2 expression in response to either LPS+IFNγ or IL-4 treatment (Fig. 6*C*). The involvement of FIH in the regulation of *Arg1* expression was further tested in FIH^+/+^ and FIH^Δ1-2/Δ1-2^ BMDMs following *M. bovis* BCG infection. FIH deficiency potentiated ARG1 expression in BCG-infected BMDMs, whereas, under the same conditions, FIH status has minimal impact on the protein expression levels of NOS2 (Fig. 6*D*). Overall, these results identify FIH as a negative regulator of Arginase expression in macrophages in response to external stimuli such as cytokines and infection.

Given that increased ARG1-expressing TAMs were seen in FIH MKO mice (Fig. 4*F*), we hypothesized that signals secreted from cancer cells, such as LLC cells, may influence *Arg1* expression in an FIH-dependent way. To confirm, we isolated TAMs from LLC tumors grown in FIH^+/+^ and FIH^Δ1-2/Δ1-2^ mice using MACS, and then measured mRNA expression levels of a number of known HIF-regulated targets including *Arg1*, *Vegfa, Il-6*, and *Nos2* (25, 38, 39) (Fig. 6*E*). Reassuringly, significant reduction of FIH was seen in TAMs isolated in FIH^Δ1-2/Δ1-2^ mice. Elevated *Arg1* expression was observed in LLC-TAMs from FIH^Δ1-2/Δ1-2^ mice (FIH^+/+^ vs. FIH^Δ1-2/Δ1-2^: 0.5352 vs. 0.9450, *P* = 0.0310). Under the same conditions, the impact of FIH gene status in LLC-TAMs on the expression levels of *Il6*, and *Nos2* did not reach statistical significance.

To investigate whether FIH can regulate myeloid *Arg1* expression in response to signals secreted by tumor cells, we treated FIH^+/+^ and FIH^Δ1-2/Δ1-2^ BMDMs with media that were conditioned by B16 or LLC cell culture supernatants (tumor cell-conditioned medium, TCM). Compared with B16 TCM, LLC TCM was a much more potent inducer for *Arg1* expression in BMDMs. FIH deletion resulted in further enhancement of *Arg1* mRNA expression in BMDMs treated with LLC TCM but not B16 TCM (Fig. 6 *F*, *Left*; relative expression to γ-actin: FIH^+/+^ vs. FIH^Δ1-2/Δ1-2^ 0.02 vs. 0.08, *P* = 0.015, *t* test). Similar enhancement was also seen at ARG1 protein level (Fig. 6 *F*, *Right*). The effect of FIH on ARG1/*Arg1* expression (both protein and mRNA level) was not specific to LLC. When we treated BMDMs with supernatants of murine colorectal carcinoma CT26 cells, again we noted enhanced induction of *Arg1* in the absence of FIH (*SI Appendix*, Fig. S6*B*; FIH^+/+^ vs. FIH^Δ1-2/Δ1-2^ 0.128 vs. 0.293, *P* = 0.047, *t* test). When co-cultured with CT26 cells, FIH^Δ1-2/Δ1-2^ BMDMs showed enhanced expression of ARG1 by Western blotting (*SI Appendix*, Fig. S6*C*). Finally, expression of FIH mRNA in BMDMs was induced by LPS+IFNγ, BCG, LLC TCM but not IL-4 treatment (*SI Appendix*, Fig. S6 *D*–*F*). These results suggest that one of the mechanisms by which FIH may suppress a tumor promoting immune environment is through its ability to repress *Arg1* expression in macrophages in response to secreted signals from tumor cells in vitro and in vivo. Reciprocally, FIH expression is also under the dynamic regulation of these environmental cues.

### FIH Deletion Promotes Macrophage Chemotaxis In Vitro and Enhances Tumor Infiltration of ARG1-Expressing Cells In Vivo.

One of the most well-known functions of macrophages is their ability to internalize large particles, known as phagocytosis (40). This process involves ligand–receptor interaction and actin cytoskeletal remodeling. We tested the role of FIH on phagocytosis of zymosan particles using FIH^+/+^ and FIH^Δ1-2/Δ1-2^ BMDMs and found that FIH status had no detectable impact on phagocytosis under normoxia or hypoxia (*SI Appendix*, Fig. S7*A*).

**Fig. 7.**
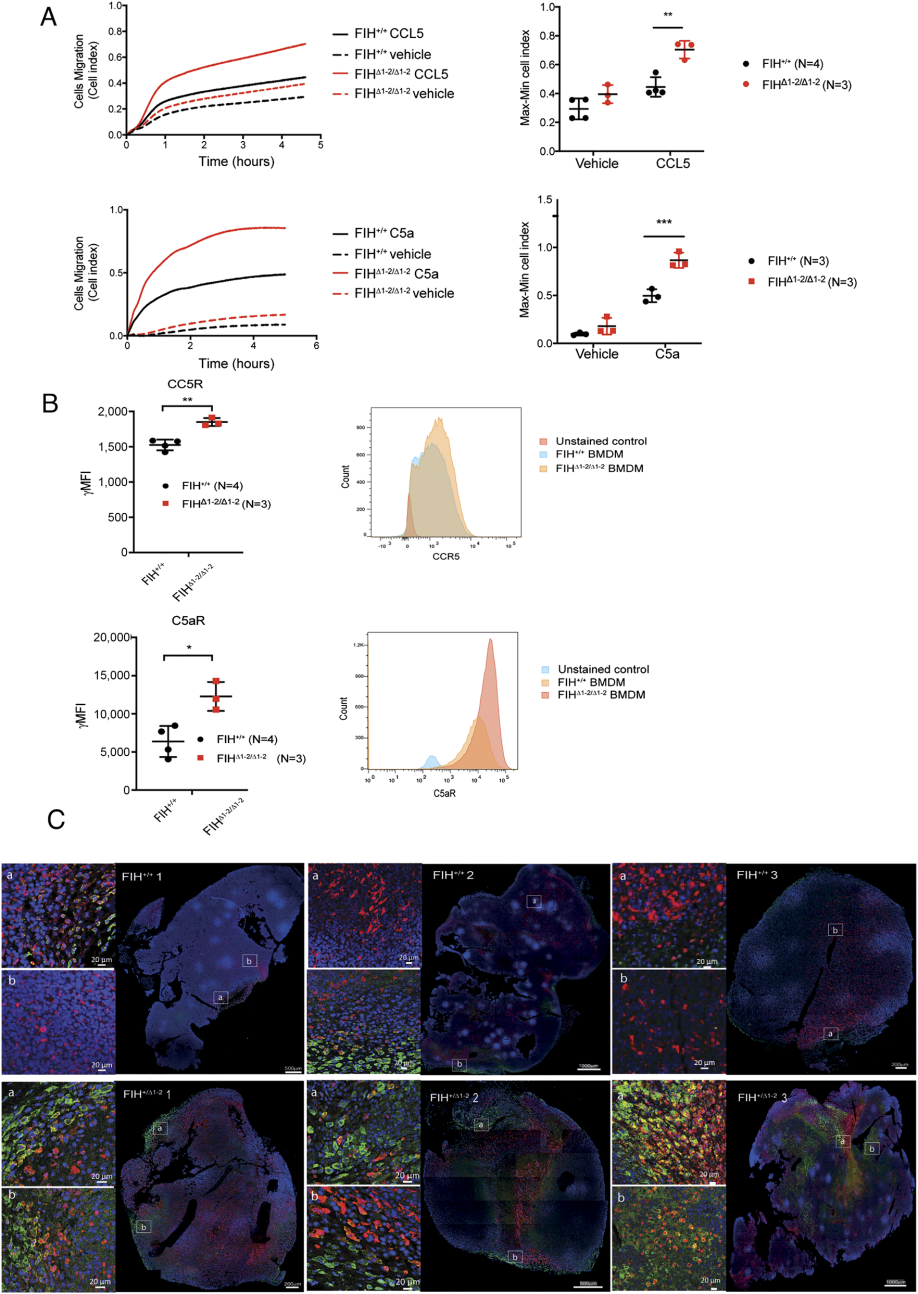
Loss of FIH promotes macrophage migration toward CCL5 and C5a and promotes ARG1+, F4/80+, and F4/80+/ARG1+ M2-like macrophage infiltration in LLC tumors. (*A*) Migration of BMDMs derived from FIH^+/+^ and FIH^Δ1-2/Δ1-2^ mice toward CCL5 (10 ng/mL; FIH^+/+^ N = 4, FIH^Δ1-2/Δ1-2^ N = 3) and C5a (10 ng/mL; FIH^+/+^ N = 3, FIH^Δ1-2/Δ1-2^ N = 3) was recorded by xCELLigence RTCA-DP instrument. Migration traces and max-min analyses of the traces are shown. (*B*) Expression levels of CCL5 receptor (CCR5) and C5a receptor (C5aR) in FIH^+/+^ (N = 4) and FIH^Δ1-2/Δ1-2^ (N = 3) BMDMs as determined by flow cytometry. gMFI, geometric mean fluorescence intensity. Horizontal lines and error bars represent mean ± SD. * indicates *P* < 0.05, ** indicates *P* < 0.01, and *** indicates *P* < 0.001, by two-tailed *t* tests. (*C*) Whole section scanning by confocal microscopy showing dual immunofluorescence staining of ARG1 (red), F4/80 (green), and DAPI (blue) in LLC tumors grown in FIH^+/+^ and FIH^+/Δ^^1-2^ mice (three replicates shown for each genotype, from three different mice). Zoomed-in regions (Scale bar, 20 µm) correspond to the adjacent white box outline on the main image. (Scale bar, 200, 500 or 1,000 µm, as indicated.)

Tumor cells are known to produce growth factors and chemokines to recruit macrophages (41). We next tested the impact of FIH on the directional migration of macrophages toward chemokine CCL5, which is known to be associated with an altered myeloid or T cell involvement in melanoma (42–44). We also tested C5a, which acts as a strong chemoattractant during infection, sepsis, and chronic inflammation (45). Macrophage migration was monitored with a real-time cell analysis (RTCA)-dual purpose (DP) system using cell invasion and migration (CIM) plates. Both CCL5 and C5a elicited robust migration of BMDMs. Compared to FIH^+/+^ BMDMs, FIH^Δ1-2/Δ1-2^ BMDMs demonstrated enhanced migration toward CCL5 and C5a (Fig. 7*A*).

Chemotaxis is initiated by interaction with a corresponding receptor expressed on the cell surface (46). FACS analysis of FIH^+/+^ and FIH^Δ1-2/Δ1-2^ BMDMs revealed that increased expression levels of chemokine receptors, C-C chemokine receptor type 5 (CCR5) and complement component 5a receptor 1 (C5aR), were detected in FIH^Δ1-2/Δ1-2^ BMDMs compared to that found in FIH^+/+^, BMDMs (CCR5: fold change 1.16, *P* = 0.0042, *t* test and C5aR: fold change 1.93, *P* = 0.0115, *t* test) (Fig. 7*B*). Increased chemokine receptor expression might explain why FIH-deficient macrophages may be more active than FIH-competent macrophages in migrating toward chemokine secreting cells such as tumor cells.

Since FIH deficiency enhances ARG1/*Arg1* expression in macrophages and promotes chemotaxis, we investigated whether FIH-deficient macrophages are more likely to infiltrate tumors to form tumor promoting TAMs in vivo. Six LLC tumors derived from three pairs of FIH^+/+^ and FIH^+/Δ^^1-2^ mice were used to examine the expression of ARG1. Immunohistochemistry results shown in *SI Appendix*, Fig. S7*B* illustrated that there is a clear enrichment of ARG1-expressing cells in LLC tumors derived from FIH^+/Δ^^1-2^ mice compared to those derived from FIH^+/+^ mice. Under the same conditions, the expression levels of VEGF, a known HIF target, are comparable among all six LLC tumors examined, consistent with previous findings that FIH status has minimal impact on the expression levels of VEGF (*SI Appendix*, Fig. S7*C*).

To provide further evidence that FIH deficiency can induce *Arg1* expression in macrophages, we also carried out double immune-fluorescent staining of ARG1 and F4/80 in LLC tumors derived from same set of LLC tumors as that shown in *SI Appendix*, Fig. S7 *B* and *C*. Comparing the LLC tumors derived from FIH^+/Δ^^1-2^ vs. FIH^+/+^ mice, the results in Fig. 7*C* showed that in addition to a large enrichment of F4/80 expressing macrophages and ARG1-expressing cells, we detected a significantly higher number of cells co-expressing both F4/80 and ARG1 in LLC tumors derived from FIH^+/Δ1-2^ mice vs. FIH^+/+^ mice. As injected LLC cells have similar genetic make-up, the detected difference in the tumor infiltration by ARG1+, F4/80+, and F4/80+/ARG1+ M2-like macrophages in FIH^+/+^ vs. FIH^+/Δ1-2^ mice reflects the interactions between the tumor cells and the TME. The external signal from TME influences LLC growth in this “Outside-In” tumor model. Together, the results shown in this study illustrate that FIH status controls immune homeostasis, and that FIH deficiency in the host can create a tumor-supportive TME to promote tumor growth.

## Discussion

One of the hallmarks of aging is immune dysfunction, which contributes to reduced surveillance and suppression of cancer cell growth (28). This is the first study to identify FIH, factor-inhibiting HIF, as a haploinsufficient tumor suppressor in mice under physiological aging conditions. Using a combination of biochemical techniques, spontaneous tumor models, and a subcutaneously implanted tumor model in syngeneic FIH-competent and -deficient mice, we demonstrated that FIH maintains immune homeostasis and suppresses tumor growth, partly through its ability to repress ARG1/*Arg1* expression in macrophages.

FIH deficiency may not only affect primary tumorigenesis, but also tumor progression. The latter is demonstrated by the increased incidence of extra-nodal B cell lymphomas in FIH-deficient animals, especially in the lungs (Fig. 1*D*). Given that all mice with pulmonary B cell lymphoma also harbor B cell lymphomas in other tissues (*SI Appendix*, Table S1), it is possible that the involvement of lung tissues is a manifestation of metastasis. While detailed molecular analysis is warranted to determine the clonality, it is widely accepted that, in clinical pathology, lymphoma presented at multiple sites are of the same clone until proven otherwise (47). Why FIH deficiency enhances the propensity of pulmonary dissemination of B cell lymphomas is not clear. The reduced expression of TGF-β, a cytokine which has been shown to induce the senescence of B cell lymphoma cells (29) (Fig. 2*D*), could be one of the underlying causes. Interestingly, FIH status does not affect the overall survival of mice (Fig. 1*A*). This is likely due to the indolent features of low-grade B cell lymphomas, which occurred at a higher proportion in FIH-deficient animals (Fig. 1*E*).

Although hypoxia and HIF signaling play key roles in B cell development through their ability to regulate metabolism, migration, and antibody class switching (48), B cell lymphoma development is an extremely complex process. It is almost impossible to conclude whether cell-intrinsic, -extrinsic, or combined pathways are responsible for the development of spontaneous tumors, B cell lymphomas in particular, in the germline deleted FIH mice. Future studies are needed to elucidate the molecular mechanisms by which FIH suppresses B cell lymphomagenesis. Nonetheless, B cell–specific deletion of HIF1α, but not HIF2α, caused reduction in the number of IL10 producing B cells, consistent with *Il10* as a transcription target of HIF (49). Notably in our study, FIH status did not affect *Il10* mRNA expression (Fig. 2*D*) and the observed tumor suppressive function of FIH is more HIF2α-dependent than HIF1α, suggesting that FIH might suppress B-cell lymphoma via an *Il10*-independent pathway.

In the human cancer genome, FIH mutation is a rare event (50, 51). Only a handful of studies have reported deletion of the chromosomal region containing *HIF1AN* (encoding FIH) in glioblastoma multiforme (52), prostate cancers (53), and a small set of pediatric T cell acute lymphoblastic leukemias (54). This is consistent with our finding that FIH could exert its tumor suppressive function via a cell-extrinsic pathway without accumulating mutations and being selected for in tumor cells. First, a lack of FIH gene dosage effect on B cell differentiation and proliferation in young adult mice with global deletion of FIH in early embryonic development (*SI Appendix*, Fig. S2) suggests FIH deficiency in B cells alone may not be sufficient to alter B cell functions. Second, Haines et al have demonstrated lymphomas to be the neoplasm of highest incidence in aging C57BL/6; 129 mice (55). Third, the initiation of marginal zone lymphomas, the main type of low-grade B lymphomas observed in FIH-deficient animals (*SI Appendix*, Table S1) is recognized as a consequence of chronic inflammation or infection (56). Consistent with these findings, compared to FIH wild-type mice, cytokine expression levels and immune cell compositions are only altered in aging FIH-deficient mice with the same genetic background (C57BL6) (Fig. 2 *C* and *D*). All these suggest, but do not prove, that FIH may have a cell-extrinsic tumor suppressive function under physiological conditions.

The non-cell autonomous effect of FIH in tumor suppression is mostly evident in the syngeneic tumor models using LLC and B16 melanoma cells (Figs. 3 and 4). LLC and melanoma (B16) cell lines are highly aggressive cancer cell lines with rapid tumor growth. It is remarkable that heterozygous or homozygous deficiency of FIH in the host, either in all cell types or in myeloid cells, can exhibit a clearly detectable impact on tumor growth in a large number of examined mice. As the impact of FIH on immune cell function increases with age, one would expect the differences in the tumor suppressive function of FIH to be greater in aged mice (>1 y old). The detected impact of FIH deficiency on tumor growth in young mice is likely to underestimate the tumor suppressive potential of FIH. The notion that the growth rate of the same subcutaneously implanted tumor cells is largely influenced by their host environment (FIH-competent or -deficient TME) is an exemplar of the Outside-In tumor model; how outside signals from the TME can influence the behavior of injected tumor cells.

The finding that myeloid-specific deletion of FIH alone is not sufficient to promote spontaneous tumorigenesis (Fig. 4*B*) nor to disturb the immune cell compositions in the spleen and liver tissues of aged mice under normal physiological conditions (*SI Appendix*, Fig. S4 *E* and *F*) illustrates that under the normal aging process, FIH deficiency in myeloid cells is not sufficient to induce tumor growth. The tumor supporting TME can only be created when tumor driving signals and tumor initiating cells are present. Therefore, it is likely that the observed spontaneous tumors in germline-deleted FIH mice are caused by a combination of cell-intrinsic and -extrinsic pathways.

In the subcutaneous syngeneic tumor model, the signals secreted from tumor cells, e.g., in TCM from LLC cells and CT26 cells, induce ARG1/*Arg1* expression in the host myeloid cells. FIH deficiency enhances the expression of a defined gene set in macrophages that includes *Arg1* and the receptors of CCR5 and C5a. As a result, FIH-defective myeloid cells can migrate toward chemokine secreting tumor cells to create a tumor promoting TME by way of forming a “great wall” which prevents T cell infiltration (57–59). However, the tumor-supportive TME is not only created by myeloid cells; other cell types such as T cells also play key roles. Although TCM from B16 cells failed to induce ARG1/*Arg1* expression in myeloid cells, an FIH-defective host environment can still create a tumor promoting TME, for example, via interactions between B16 cells and various FIH-defective cells, including T cells. In the germline-deleted FIH mice, FIH-defective T cells might contribute to B16 tumor growth. In FIH-MKO mice, FIH-defective myeloid cells could interact with FIH-competent T cells to control their function and their ability to infiltrate tumors. In agreement with this, we observed a reduction in the number of tumor-infiltrating T cells in tumors derived from FIH-defective mice vs. FIH WT mice. Moreover, a reduction in tumor-infiltrating CD4 and CD8 T cell populations was also observed in myeloid-specific FIH deficient mice (Figs. 3*D* and 4*G*). A recent study showed that in an adaptive T cell therapy setting, exogenously expressed FIH-insensitive HIF2α increases antitumor properties of CD8 T cells (60). Future studies of the FIH/HIF interaction in other immune cells, especially lymphocyte lineages, will be of interest to further delineate mechanistic details underlying the tumor-promoting effects of FIH deficiency.

The immune regulatory effect of FIH is not limited to the tumor setting but is also present in other inflammatory conditions such as infection. We demonstrated that BCG infection induces FIH expression (*SI Appendix*, Fig. S6*E*), suppressing the induction of *Arg1* in BMDMs (Fig. 6*D*). A recent study of FIH in the colonic epithelium showed FIH deficiency can attenuate chronic colitis induced by azoxymethane/dextran sodium sulfate (61), suggesting that inhibiting FIH could benefit patients. A series of 2-oxoglutarate derivatives that selectively modulate FIH activity have been identified, enabling researchers to evaluate the therapeutic efficacy of targeting FIH in vivo (62). The identification of FIH as a key regulator of immune homeostasis and a tumor suppressor under physiological conditions in vivo provides vital information which will guide the future development and application of FIH modulators to treat various inflammatory conditions such as infection, autoimmune disorders, and cancer.

## Materials and Methods

Complete experimental methods are described in *SI Appendix*, *Materials and Methods*.

### Mouse Colonies.

FIH, HIF1α, and HIF2α transgenic mice were generated in Prof. Peter Ratcliffe's laboratory. FIH transgenic mice were originally generated by InGenious Targeting Laboratory (Ronkonkoma) on a C57BL/6 × 129/SvEv background. Exon 1 and exon 2 of *Hif1an* (encoding FIH) was flanked by LoxP sites and deleted at an early embryonic stage by crossing with C57BL/6 Sox2-Cre mice. HIF1α^fl/fl^ and HIF2α^fl/fl^ (where “fl” denotes the floxed allele) conditional knockout mice have been described previously and were obtained from the following sources (24, 63).

For syngeneic tumor studies, mice with the FIH knockout allele were backcrossed in a C57BL/6 background for eight generations before the administration of tumor cells of C57BL/6 origin. To generate myeloid-specific FIH knockout (FIH MKO) mice, mice harboring the FIH^fl/fl^ alleles were crossed with Lysozyme 2 (LysM)-Cre animals on a C57BL/6 background. To generate HIF1α- and HIF2α-MKO (myeloid-specific knockout) mice, LysM-Cre mice were crossed with HIF1α^fl/fl^ and HIF2α^fl/fl^ conditional knockout mice, respectively. LysM-Cre animals were purchased from the Jackson Laboratory.

### Flow Cytometry.

Flow cytometry analysis of murine tissues was undertaken on an LSRFortessa X-20 cell analyzer, and FACS data were analyzed using FlowJo v10. Sample preparation and antibody staining are detailed in *SI Appendix*, *Materials and Methods*.

### Protein Analysis.

Protein analysis was carried out by SDS-PAGE (Mini-PROTEAN® Tetra Cell system) and Western blotting onto a nitrocellulose membrane (Whatman) using a wet transfer system (Hoefer) and revelation was carried out by enhanced chemiluminescence (GE Healthcare). Detailed methods, including antibodies and incubation conditions, are described in *SI Appendix*.

## Supplementary Material

Appendix 01 (PDF)

## Data Availability

Western blots, histopathology images, immunofluoresence images, FACS data, tumor volume and weights, RNA sequencing data, RT qPCR analysis, macrophage migration traces, and cell viability assay data have been deposited in Mendeley (64, 65).
