# Supplementary material for: Deficiency of factor-inhibiting HIF creates a tumor-promoting immune microenvironment
Source: Proc Natl Acad Sci U S A. 2024 Feb 29;121(10):e2309957121. doi: 10.1073/pnas.2309957121 (PMC10927516; doi:10.1073/pnas.2309957121)
Supplement: Supplementary file 1 — Appendix 01 (PDF) [file pnas.2309957121.sapp.pdf]

PNAS

Supporting Information for

Deficiency of Factor Inhibiting HIF creates a tumor-promoting immune microenvironment.

Jingyi Ma[1, 14], , Khatoun Al Moussawi[1], Hantao Lou[1], Hok Fung Chan[1], Yihua Wang[1,8], Joseph Chadwick[1], Chansavath Phetsouphanh[1,3], Elizabeth A. Slee[1], Shan Zhong[1], Thomas M. Leissing[1], Andrew Roth[1,11,12,13], Xiao Qin[1,9], Shuo Chen[1], Jie Yin[1], Indrika Ratnayaka[1], Yang Hu[1], Pakavarin Louphrasitthiphol[1], Lewis Taylor[4], Paulo J. G. Bettencourt[5,10], Mary Muers[1], David R Greaves[4], Helen McShane[5], Robert Goldin[6], Elizabeth J. Soilleux[7], Mathew L. Coleman[2], Peter J. Ratcliffe[1] and Xin Lu[1]*

*Corresponding author: Xin Lu
Email: xin.lu@ludwig.ox.ac.uk

This PDF file includes:

Supplementary Figures S1 to S7
Materials and methods
SI references
Acknowledgements
Supplementary Tables 1 to 11

Supplementary Figure 1

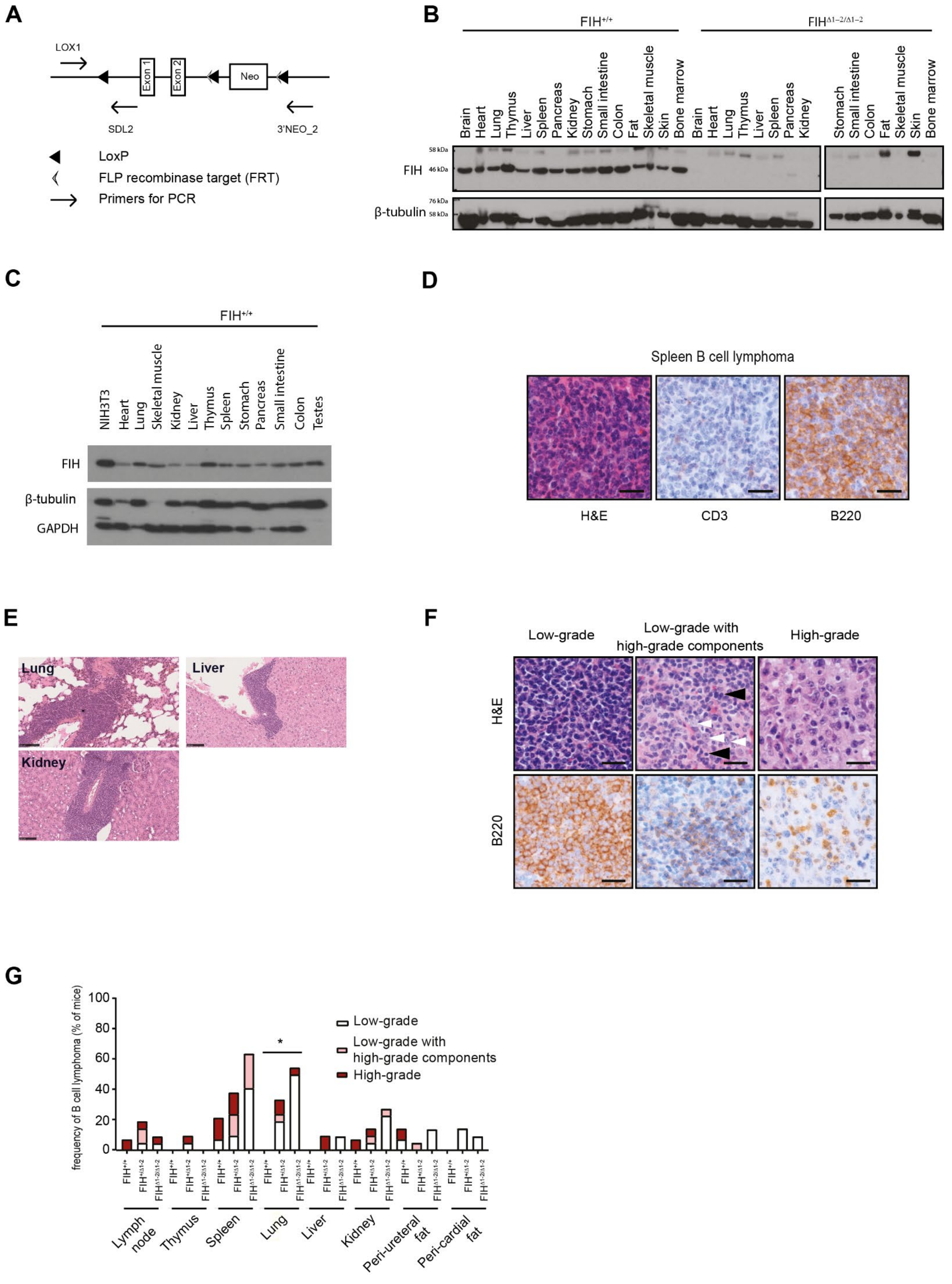

Fig. S1. Reduced FIH expression led to increased lymphomagenesis

(A) Schematic representation of the targeted FIH allele. **(B)** Western blots showing FIH expression in indicated tissue lysates prepared from $FIH^{+/+}$ and $FIH^{\Delta 1\text{-}2/\Delta 1\text{-}2}$ mice with β-tubulin being the loading control. **(C)** FIH expression in indicated tissues from $FIH^{+/+}$ mice compared with a mouse fibroblast cell line NIH3T3 by Western blotting. Both GAPDH and β-tubulin were used as loading controls showing the variation in their expression levels. **(D)** Representative haematoxylin and eosin (H&E) and immunohistochemistry (IHC) staining showing a case of B cell lymphoma in the spleen found in an $FIH^{\Delta 1\text{-}2/\Delta 1\text{-}2}$ mice. Scale bar, 25 μm. **(E)** Representative H&E staining with B cell lymphomas in non-lymphoid tissues: lung, liver, and kidney. Scale bar, 100 μm. **(F)** Representative H&E and IHC staining showing examples of splenic B cell lymphoma of low-grade, low-grade with high-grade components and high-grade lymphoma. Black arrows point to cells with low-grade features and white arrows indicate cells with high-grade features. Scale bar, 25 μm. **(G)** Frequencies of histologically diagnosed low-grade, low-grade with high-grade components, and high-grade B cell lymphomas in indicated tissues. Frequencies were obtained by dividing the number of mice with indicated type of lymphoma by the total number of mice of this genotype. A higher frequency of mice with low-grade B cell lymphoma in the lung was observed in the $FIH^{\Delta 1\text{-}2/\Delta 1\text{-}2}$ mice ($FIH^{+/+}$ vs $FIH^{\Delta 1\text{-}2/\Delta 1\text{-}2}$ mice: 0 vs 50%, p=0.0020 by χ^2 test).

Supplementary Figure 2

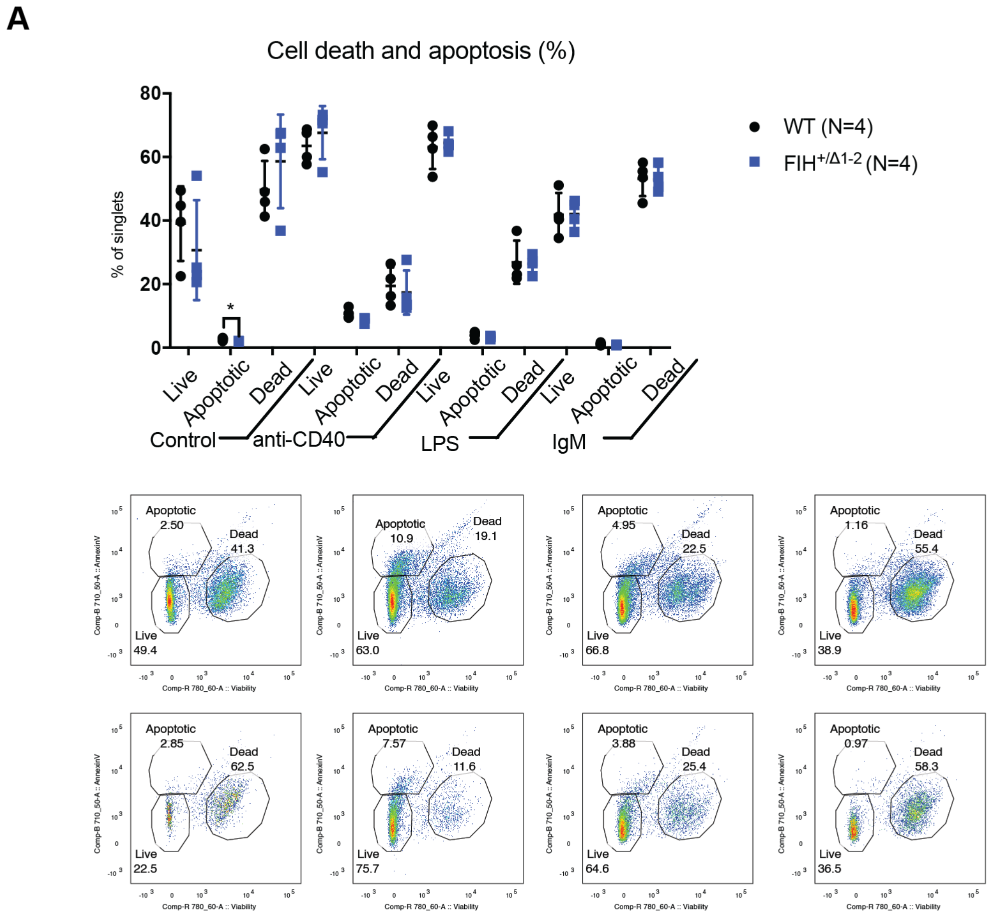

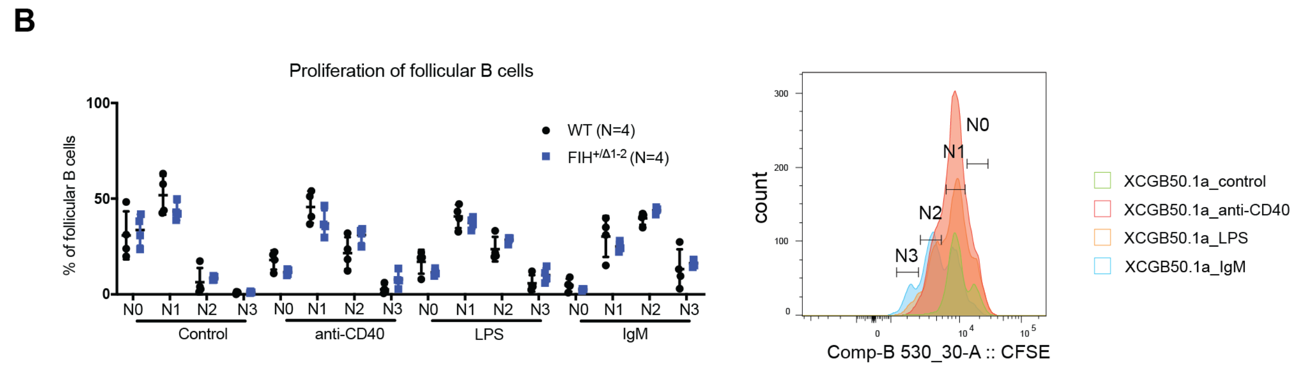

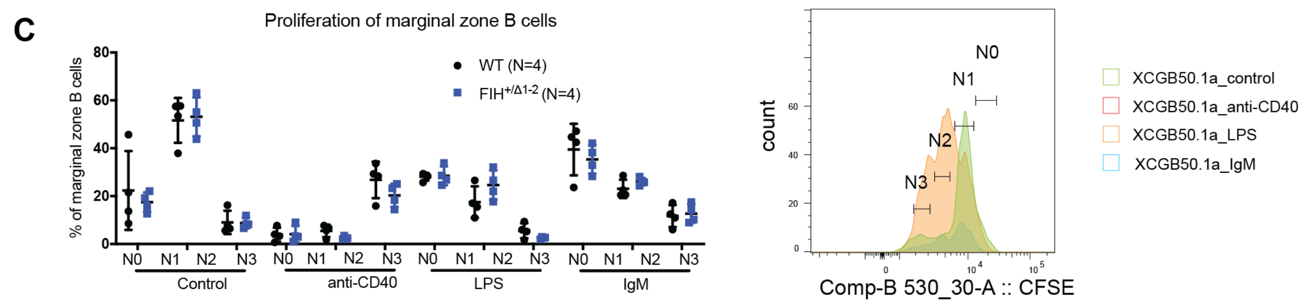

D

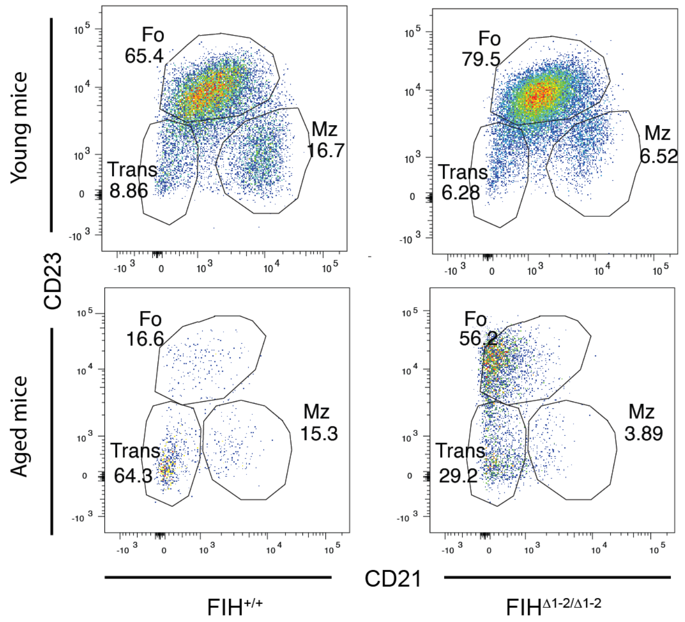

Young mice
Aged mice
CD23
CD21
Fo
65.4
Mz
16.7
Trans
8.86
Fo
79.5
Mz
6.52
Trans
6.28
Fo
16.6
Mz
15.3
Trans
64.3
Fo
56.2
Mz
3.89
Trans
29.2
FIH+/+
FIHΔ1-2/Δ1-2

E

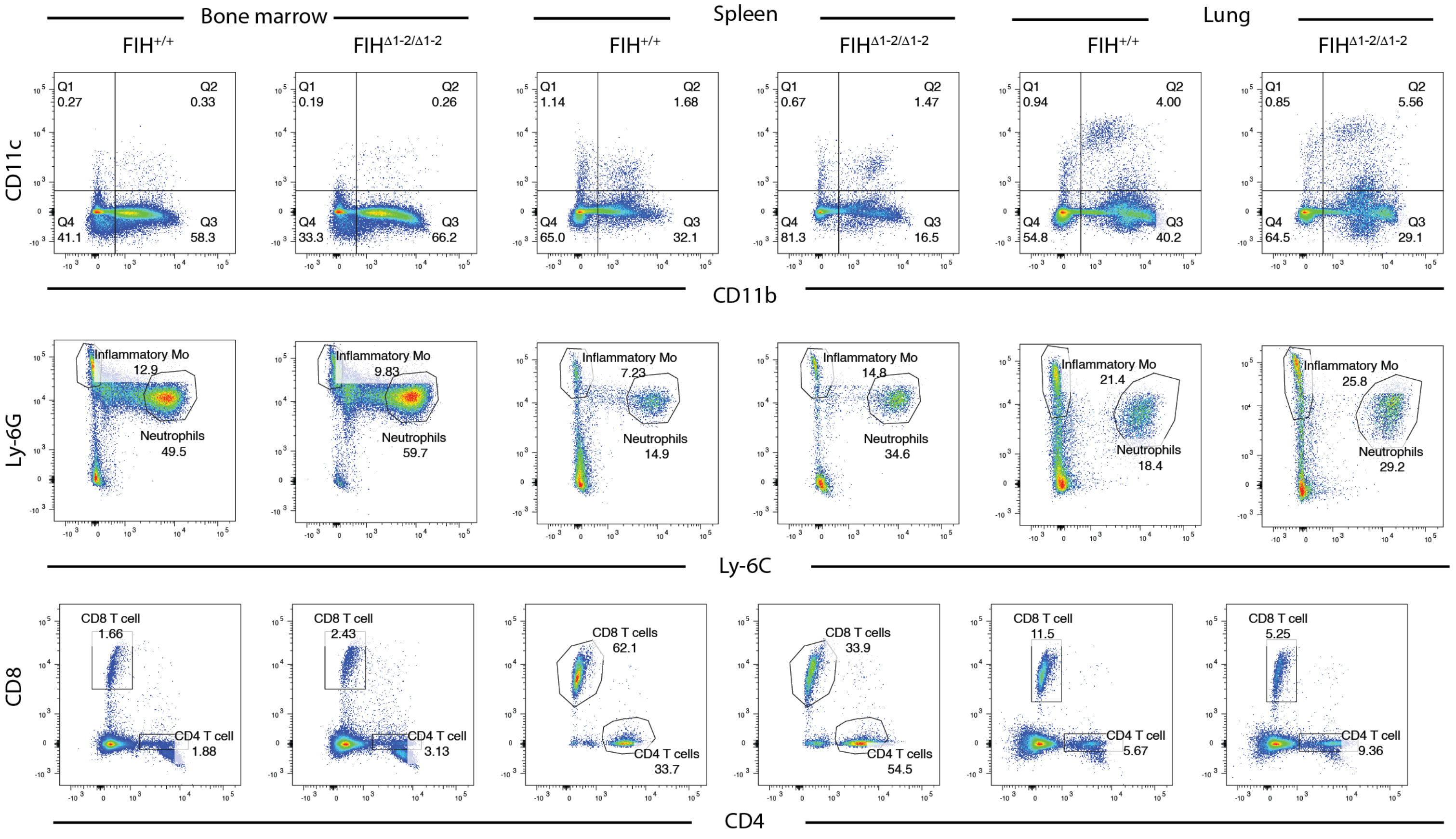

Bone marrow
Spleen
Lung
FIH+/+
FIHΔ1-2/Δ1-2
CD11c
CD11b
Q1 0.27 Q2 0.33 Q4 41.1 Q3 58.3
Q1 0.19 Q2 0.26 Q4 33.3 Q3 66.2
Q1 1.14 Q2 1.68 Q4 65.0 Q3 32.1
Q1 0.67 Q2 1.47 Q4 81.3 Q3 16.5
Q1 0.94 Q2 4.00 Q4 54.8 Q3 40.2
Q1 0.85 Q2 5.56 Q4 64.5 Q3 29.1
Ly-6G
Ly-6C
Inflammatory Mo 12.9 Neutrophils 49.5
Inflammatory Mo 9.83 Neutrophils 59.7
Inflammatory Mo 7.23 Neutrophils 14.9
Inflammatory Mo 14.8 Neutrophils 34.6
Inflammatory Mo 21.4 Neutrophils 18.4
Inflammatory Mo 25.8 Neutrophils 29.2
CD8
CD4
CD8 T cell 1.66 CD4 T cell 1.88
CD8 T cell 2.43 CD4 T cell 3.13
CD8 T cells 62.1 CD4 T cells 33.7
CD8 T cells 33.9 CD4 T cells 54.5
CD8 T cell 11.5 CD4 T cell 5.67
CD8 T cell 5.25 CD4 T cell 9.36

Fig. S2. Reduced FIH expression does not affect B cell proliferation *in vitro*

Splenic B cells of $FIH^{+/+}$ (N=4) and $FIH^{+/\Delta 1\text{-}2}$ (N=4) mice were enriched by magnetic-activated cell sorting (MACS) and stimulated by anti-CD40 monoclonal antibody, LPS, or IgM for 48 hours. (A) Percentages of live (Annexin V^-, Viability dye^-), apoptotic (Annexin V^+, Viability dye^-), and dead (Annexin V^{Int}, Viability dye^+) splenocytes after stimulation analysed by flow cytometry. Representative FACS plots are shown below. (B and C) Proliferation of follicular B cells ($CD21^{Int}CD23^+$) (B) and marginal zone B cells ($CD21^+CD23^{Int}$) (C) by CFSE dilution assay. Horizontal lines and error bars represent mean ± SD. * indicates $p<0.05$ by two-tailed *t*-tests. Exemplar replicating profiles are shown on the right, with peaks corresponding to each round of cell division. XCGB50.1a corresponds to samples from a single WT mouse. N indicates the generation, i.e., N0 indicates a population which has not yet undergone mitosis. (D) Representative FACS plots from flow cytometry analysis of B cell subsets in the spleens of 18-week-old (young) and 104-week-old (aged) $FIH^{+/+}$ mice (N=4) and $FIH^{\Delta 1\text{-}2/\Delta 1\text{-}2}$ mice (N=3) showing gating of follicular B cells, marginal zone B cells, and transitional B cells on CD19+ CD220+ cells. (E) Representative FACS plots from flow cytometry profiling of immune cell subtypes in the indicated tissues of 104-week-old (aged) $FIH^{+/+}$ (N=4) and $FIH^{\Delta 1\text{-}2/\Delta 1\text{-}2}$ (N=3) mice showing gating of CD11b+ CD11c- cells, neutrophils, inflammatory monocytes, and T cells.

Supplementary Figure 3

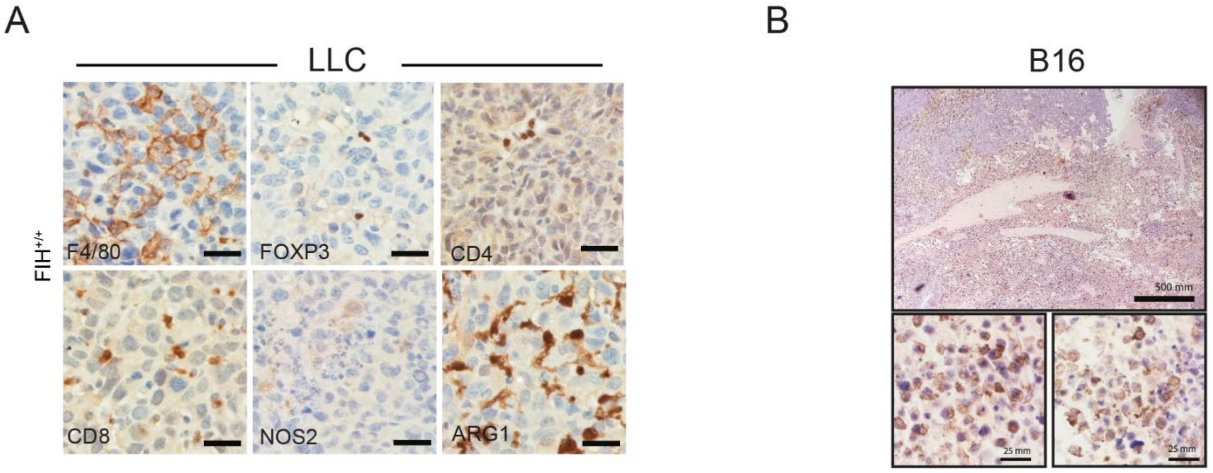

Fig. S3. Histological analysis of LLC and B16 tumors

(A) Representative IHC staining of F4/80, FOXP3, CD4, CD8, NOS2 and Arginase (ARG1) in an LLC tumor from a WT mouse. Scale bar, 50 μm. (B) $FIH^{+/+}$ (N=5), $FIH^{+/\Delta 1\text{-}2}$ (N=5), and $FIH^{\Delta 1\text{-}2/\Delta 1\text{-}2}$ (N=4) mice were subjected to subcutaneous injection of 1×10^5 B16 cells on both flanks. H&E staining of B16 tumor at the endpoint of the study (day 14) showing brown stained areas indicative of necrosis.

Supplementary Figure 4

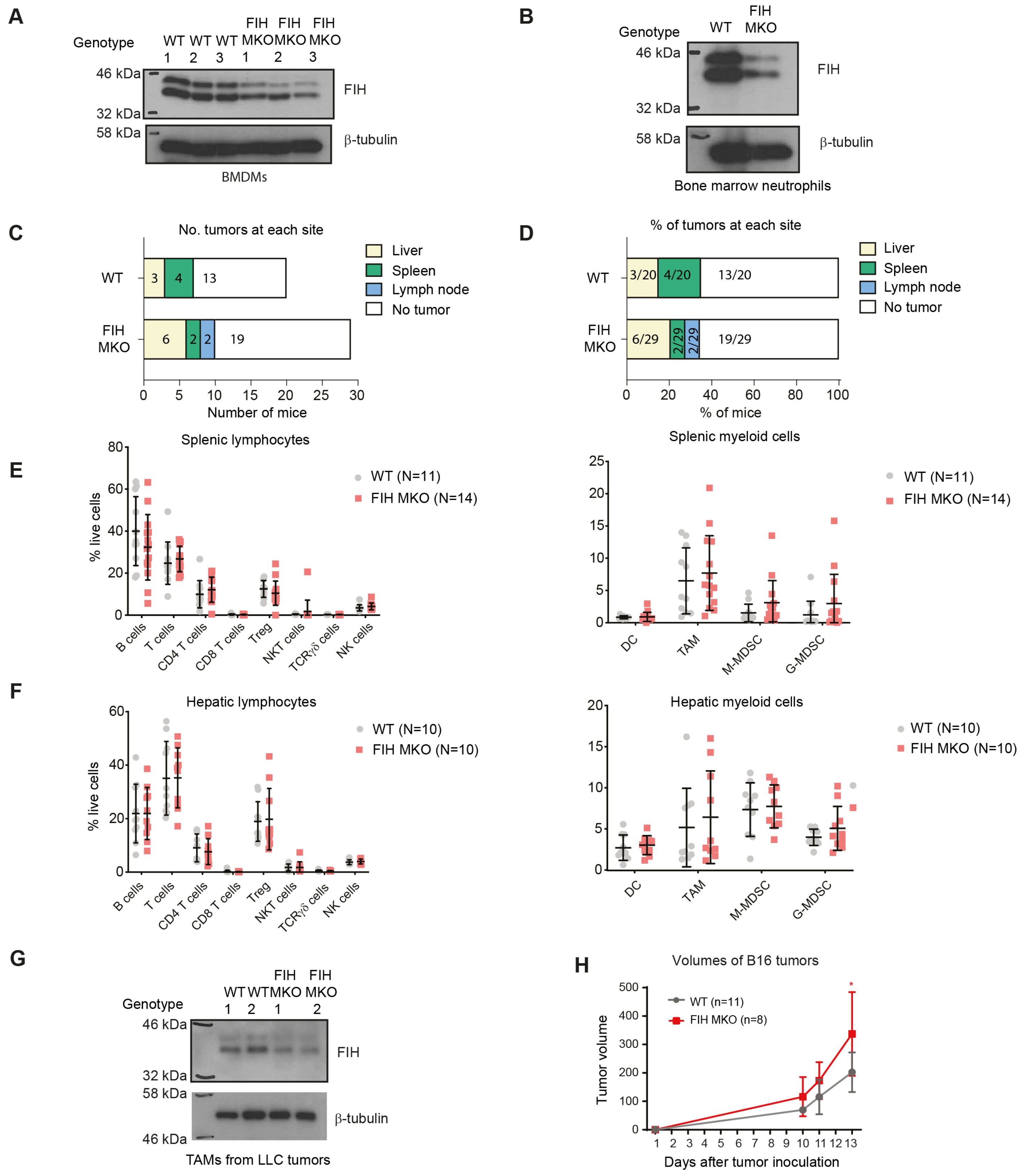

Fig. S4. Myeloid FIH expression does not affect spontaneous tumorigenesis but suppresses B16 tumor growth in young mice.

(A) Western blots showing FIH expression in bone marrow-derived macrophages (BMDMs) obtained from WT and FIH MKO animals. (B) Western blots showing FIH expression in bone marrow neutrophils in WT and FIH MKO animals. In all Western blots, β-tubulin is used as a loading control. (C and D) A cohort of WT (N=20) and FIH MKO (N=29) mice were monitored for 125 weeks. Tumor observed at various sites during the course of the study are presented as count (C) and percentage (D). (E and F) Flow cytometry profiling of immune cells (B cells, $CD4^+$ T cells, $CD8^+$ T cells, $CD11b^+$ myeloid cells, T_{reg} ($CD25^+CD4^+$), NKT cells ($CD3^+CD49^+$), γδ T cells (γδ TCR^+CD3^+), NK cells ($CD3^-CD49b^+$), DC (dendritic cells, $CD11b^-CD11c^+$), TAMs, M-MDSCs, and G-MDSCs) in the spleen (E) and liver (F) of WT and FIH MKO mice. (G) Western blots showing FIH expression in TAMs isolated from LLC tumors grown in WT and FIH MKO mice. (H) WT (N=6) and FIH MKO (N=4) mice were subjected to subcutaneous injection of 1×10^5 B16 cells on both flanks. Average volumes of B16 tumors from day 1–13. Tumor volumes, number of tumors measured and number of mice with measurable tumors at each time point are shown in Supplementary Table 5.

Supplementary Figure 5

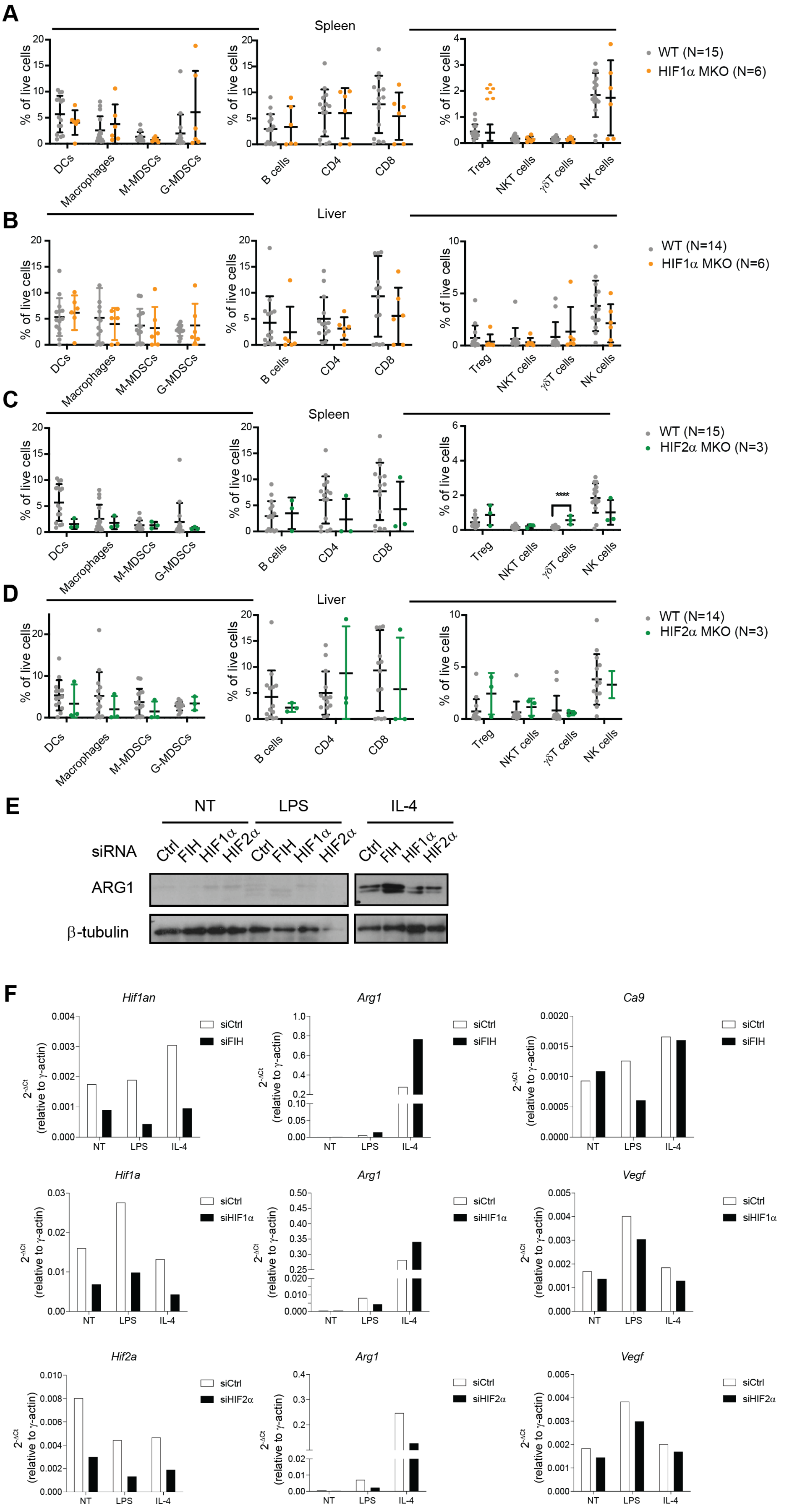

Fig. S5. Mice with HIF2α deletion in myeloid cells show increased γδT cells in the spleen

(A and B) Flow cytometry profiling of B cells, $CD4^+$ T cells, $CD8^+$ T cells, $CD11b^+$ myeloid cells, T_{reg}, NKT cells, γδ T cells, NK cells, DCs, macrophages, M-MDSCs, and G-MDSCs in (A) the spleen and (B) liver of WT (N = 15) and HIF1α MKO (N = 6) mice. (C and D) Flow cytometry profiling of immune cells in (C) the spleen and (D) liver of WT (N = 14) and HIF2α MKO (N= 3) mice. Small horizontal lines indicate the mean ± SD. **** indicates $p<0.0001$ by two-tailed *t*-test. (E and F) Murine macrophage cell line J774 cells were knocked down with indicated oligos for 48 hours and stimulated accordingly for 48 hours (E) ARG1 and β-tubulin expression was detected by Western blotting. (F) Expression of *Arg1* and other known targets of HIFs were examined by qRT-PCR. (NT: no treatment).

Supplementary Figure 6

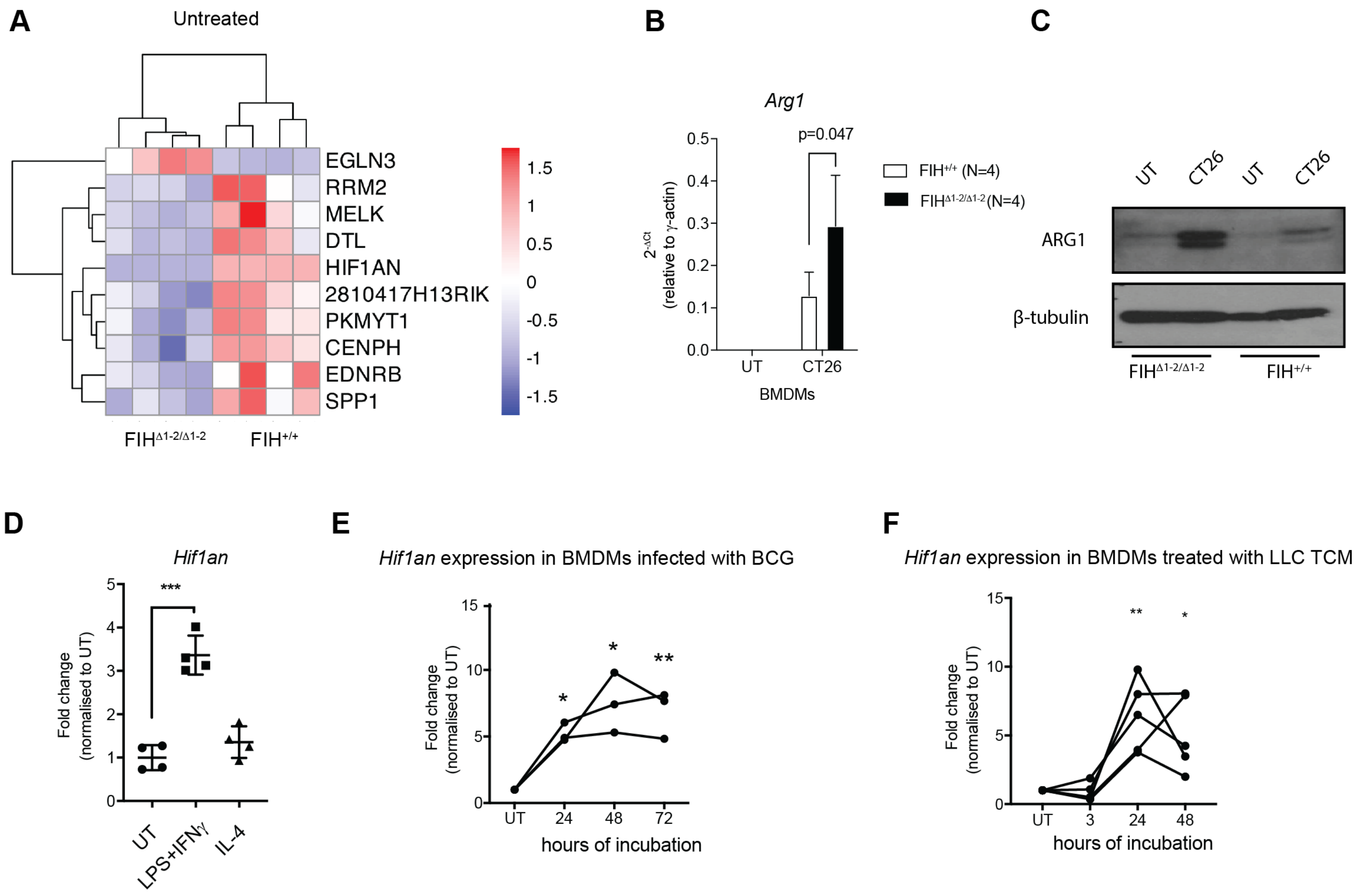

Fig. S6. ***Arg1*** **expression is affected by HIF2α status.**

(A) BMDMs harvested from $FIH^{+/+}$ (N=4) and $FIH^{\Delta 1\text{-}2/\Delta 1\text{-}2}$ (N=4) were subjected to RNA sequencing. A clustered heat map showing the differentially expressed genes (absolute log2 fold change is above 1 and FDR is less than 0.05). Colour key indicates the z-scores of normalised expression values. (B) BMDMs harvested from $FIH^{+/+}$ (N=4) and $FIH^{\Delta 1\text{-}2/\Delta 1\text{-}2}$ (N=4) were treated with the supernatants of CT26 cells for 24 hours. Arg1 expression was examined with RT-qPCR. (C) BMDMs from $FIH^{+/+}$ and $FIH^{\Delta 1\text{-}2/\Delta 1\text{-}2}$ mice were co-cultured with murine colorectal carcinoma cell line CT26 cells for 48 hours. Expression of ARG1 was detected by Western blotting. (D-F) *Hif1an* expression relative to *Actg1* (γ-actin) in WT BMDMs treated with LPS + IFNγ or IL-4 (D), BCG (E), or LLC-TCM (F) was measured by RT-qPCR. * indicates $p<0.05$ and ** indicates $p<0.01$ by two-tailed *t*-tests. UT, untreated. The bars are displayed as mean ± SD.

Supplementary Figure 7

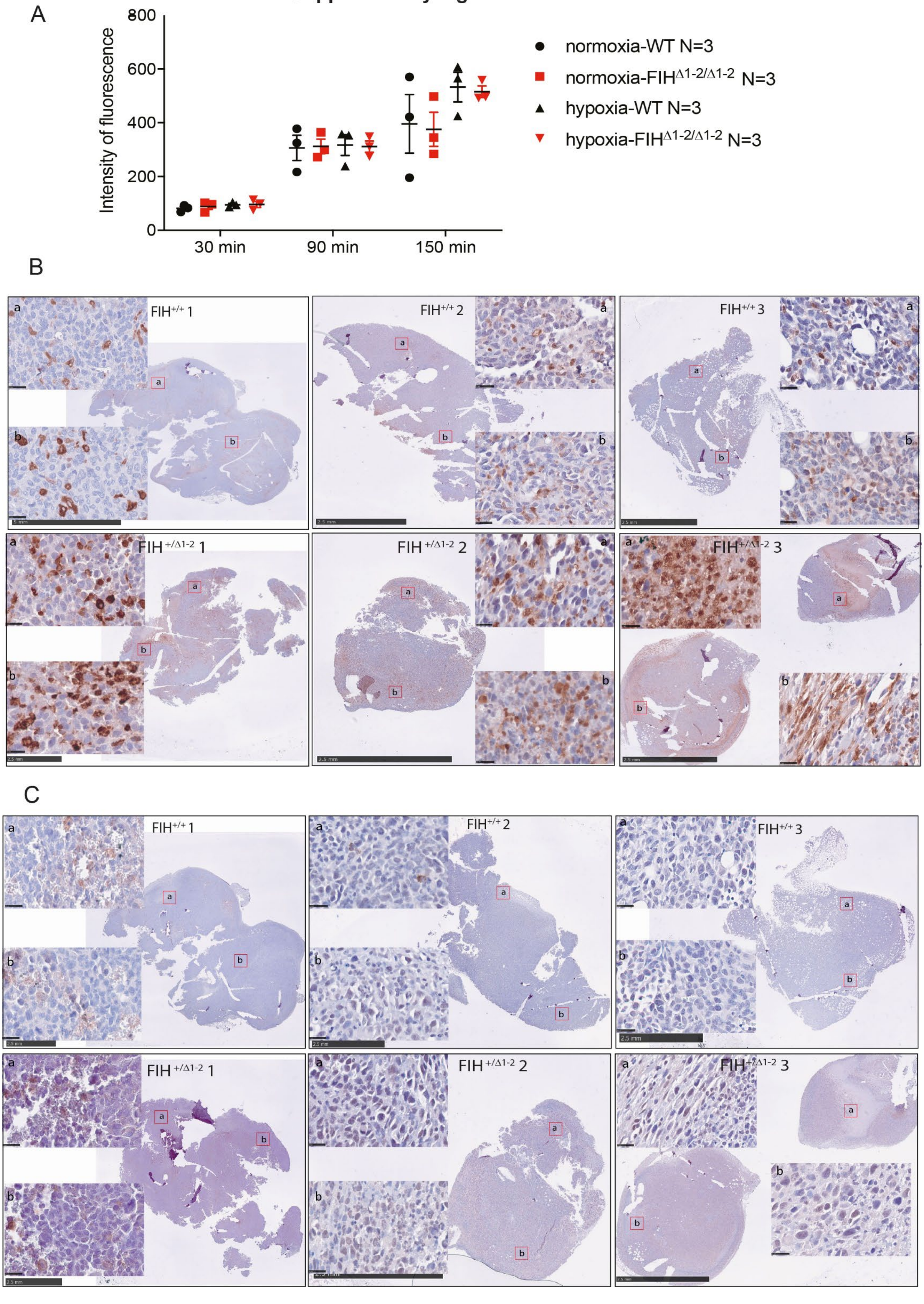

Fig. S7. FIH status does not affect phagocytosis of zymosan particles in hypoxia or normoxia conditions, but does influence the expression of ARG1 and VEGF in LLC tumors.

(A) Phagocytosis of pH-sensitive fluorescent dye-conjugated zymosan particles by BMDMs derived from $FIH^{+/+}$ (N=3) and $FIH^{\Delta 1\text{-}2/\Delta 1\text{-}2}$ (N=3) mice. Phagocytosis assays were carried out under normoxia (solid bars) or hypoxia (1% O_2, dashed bars) with indicated incubation time (from 30 min to 150 min). The bar graphs are displayed as mean ± SD. (B - C) IHC staining of ARG1 (B) and VEGF (C) in LCC tumors grown in $FIH^{+/+}$ (n=3) and $FIH^{+/\Delta 1\text{-}2}$ (n=3) mice. Scalebars in the whole slide imaging (larger images) = 2.5mm except panel B, top left box where main image scalebar = 5mm; scalebars in the insets (smaller images) = 50 μm.

Materials and Methods

Antibodies and cell lines are listed in Supplementary Tables 9–11.

Mouse colonies

Mice were housed in the Functional Genetics Facility of the Wellcome Trust Centre for Human Genetics (University of Oxford) in individually ventilated cages with food and water provided ad libitum and on a 12-hour light/12-hour dark cycle. All animal procedures were approved by the University of Oxford's ethical review committee and licensed by the UK Home Office (license number PPL 30/2862).

Spontaneous tumor analysis

Mice that showed signs of illness or visible tumors were culled and subjected to necropsy. Heart, lung, thymus, liver, pancreas, stomach, intestine, and lymph nodes were collected, and tissue histopathology was analysed in collaboration with pathologists Profs. Robert Goldin (Imperial College London) and Elizabeth J. Soilleux (University of Cambridge).

Preparation of FIH protein and antibody generation

FIH constructs d25 and d36 (the d25 construct means the protein starts from the 26th amino acid whereas the d36 starts from the 37th amino acid) were subcloned in a pET28a between a NheI and BamHI restriction enzyme cleavage site. Plasmids were transformed in E. coli BL21 (DE3) cells and a single colony was picked and grown in LB medium (100 mL) supplemented with kanamycin (30 μg/mL). The stationary starter culture was diluted 1:100 in fresh medium supplemented with the appropriate antibiotics and grown to an OD600~0.8 at 37°C prior to induction with isopropyl β-D-thiogalactopyranoside (IPTG, final concentration 0.5 mM) for 4 h at 37°C. Cells were harvested via centrifugation (10,000 g, 8 min, JA-10 rotor, Beckman Coulter). The cell pellet was stored at -80 °C. FIH containing pellets were re-suspended in 50 mM HEPES buffer (pH = 7.5, 500 mM NaCl, 5 mM imidazole) with added DNaseI from bovine pancreas (Roche) and lysed by sonication. The lysate was centrifuged (4°C, 34,000 g, 30 min, JLA1625 rotor, Beckman Coulter) and the supernatant filtered to remove cell debris. His-tagged proteins were immobilized on a His-Trap (5 mL) column, washed with low imidazole buffer (50 mM HEPES pH = 7.5 buffer supplemented with 500 mM NaCl and 20–40 mM imidazole) and eluted with a linear gradient of imidazole (max. 50

mM HEPES pH = 7.5, 500 mM NaCl, 500 mM imidazole). The N-terminal His6-tag was cleaved by the addition Thrombin (4 units per mg) overnight at 4°C. Proteases and cleaved tag were removed via SEC using a Superdex S200 column equilibrated with 50 mM Tris-HCl pH = 7.5. Completeness of the cleavage was verified by Mass Spectrometry.

Splenocytes from BALB/c mice hyperimmunised with the recombinant FIH protein d25 were fused with a non-producing mouse myeloma cell line SP2 using polyethylene glycol as a fusogen. Supernatants of selected hybrids were screened primarily using dot-blot on nitrocellulose membrane coated with FIH protein d25 and d36 followed by validation in various murine primary tissues with Western blotting.

Syngeneic tumor model

Mice with matched sex and age (8–14 weeks) were selected as experimental animals. 4×10^5 LLC cells or 1×10^5 B16 cells suspended in 100 µL of PBS were injected subcutaneously. The length and width of the tumor mass was measured with a calliper during tumor growth. Tumor volume (V) was calculated using the formula $V=(\text{length} \times \text{width}^2) \div 2$.

Haematoxylin and Eosin (H&E) staining

Tissues were collected and fixed in 10% neutral buffered formalin solution at 4°C for 24 h. Fixed tissues were processed in an Excelsior AS tissue processor (Thermo Fisher) using the routine overnight programme. Processed tissues were embedded into paraffin blocks and sectioned at 4 µm-thick. FFPE tissue sections were deparaffinised in two changes of histoclear (National diagnostics) and rehydrated in a gradient of ethanol solution (100, 90, and 70%). Slides were sequentially incubated in Harris haematoxylin for 3 min, acid alcohol (1% v/v) for 2 s, Scott's water for 30 s, and eosin for 3 min, with washes in tap water between each step. Sections were then dehydrated in a gradient of ethanol (70, 90, 100%) and two changes of histoclear before being mounted with mounting medium (Vectamount, Vector Labs).

Tissue immunohistochemistry (IHC) and immunofluorescence (IF) staining

For IHC staining on paraffin sections, slides were deparaffinised and hydrated by serial incubation with histoclear, 100% ethanol, 90% ethanol, and 70% ethanol. Sections were incubated in 3% (v/v) H_2O_2 in methanol for 10 min at RT to inactivate endogenous peroxidase. Antigenic epitopes were

retrieved by incubating the slides in boiling sodium citrate buffer (pH 6.0) for 10 min. Slides were left in the buffer to cool for 20 min at RT. Samples were blocked with 10% (v/v) normal goat serum (NGS)/PBS for 2 h at RT. Sections were then incubated with primary antibodies diluted in 5% (v/v) NGS/PBS overnight at 4 °C in a humidified chamber, followed by incubation with biotinylated secondary antibodies diluted in 5% (v/v) NGS/PBS for 1 h at RT. Slides were washed with PBS for 15 min after each incubation with antibodies. Sections were then incubated in Avidin-Biotin Complexes (ABC Reagent, Vector Labs) for 20 min at RT, washed in PBS, and applied with HRP substrate solution (DAB substrate kit, Vector Labs) till a dark brown colour was visualized. Slides were counterstained with haematoxylin, dehydrated by immersion in increasing gradients of ethanol solution, and mounted with mounting medium (Vectamount, Vector Labs).

For immunofluorescence staining on paraffin sections, slides were deparaffinised and hydrated by serial incubation with histoclear, 100% ethanol, 90% ethanol, and 70% ethanol. Sections were rinsed in water. Antigenic epitopes were retrieved by incubating the slides in boiling sodium citrate buffer (pH 6.0) for 10 min. Slides were left in the buffer to cool for 20 min at RT. Slides were washed in PBS and then permeabilized with 0.2% triton for 10 min. Samples were blocked with 10% (v/v) normal goat serum (NGS)/PBS for 1h at RT. Sections were then incubated with primary antibodies diluted in 10% (v/v) NGS/PBS overnight at 4°C in a humidified chamber. Slides were washed with PBS for 30 min followed by incubation with Alexa-Fluor secondary antibodies (1/500) diluted in 10% (v/v) NGS/PBS for 1h at RT. Slides were washed with PBS for 30 min after each incubation with antibodies then incubated with Dapi 1/500 for 10 min. Slides then washed with PBS for 5 min before mounting.

Magnetic-activated cell sorting (MACS)

Tumor-associated macrophages were enriched from syngeneic LLC tumors with an Anti-F4/80 MicroBeads UltraPure kit (130-110-443, Miltenyi Biotec). Bone marrow neutrophils and splenic B cells were enriched using mouse neutrophil isolation kit (130-097-658, Miltenyi Biotec) and B cell isolation kit (130-090-862, Miltenyi Biotec) according to the manufacturer's protocol. Briefly, single-cell suspension of the target tissue underwent magnetic labelling followed with subsequent manual cell separation using LS columns (130-042-401, Miltenyi, Biotec).

Culture of bone marrow-derived macrophages (BMDMs)

4×10^6 bone marrow cells were placed in a 10 cm petri dish containing 10 mL of macrophage growth medium, which was complete DMEM (cDMEM) containing 15% (v/v) L929-conditioned medium (LCM). Cells were kept in a humidified incubator with 5% CO_2 at 37°C. Another 5 mL of macrophage growth medium was added after 3 days of culture. For co-culture experiments, 2×10^6 BMDMs were plated on 6-well carrier plates (ThermoFisher) and 2×10^5 CT26 cells were added to the cell inserts (ThermoFisher).

In vitro stimulation of BMDMs

BMDMs were harvested, counted, and re-plated in 24-well plates with 5×10^5 cells per well in cDMEM without LCM. Cells were left quiescent overnight and stimulated the next day. BMDMs were polarised to M1 macrophages by stimulation with 5 ng/mL LPS plus 1 ng/mL IFNγ (PeproTech) for 24 hours, and to M2 macrophages by stimulation with 10 ng/mL IL-4 (PeproTech) for 24 hours. For cells stimulated with B16- or LLC-derived tumor-conditioned medium (TCM), 1 volume of TCM was mixed with 1 volume of cDMEM before application on to cells.

Infection of BMDMs with Bacillus Calmette-Guérin (BCG)

Mycobacterium bovis Bacillus Calmette Guérin (BCG) Montreal (ATCC 35735), containing EGFP cloned under the control of mycobacterial 19 kDa promoter (1) was kindly provided by Dr Rajko Reljic from St George's University of London. BCG was cultured in Middlebrook 7H9 medium (Sigma, St. Louis, MO, USA), supplemented with10% (v/v) oleic acid-albumin-dextrose-catalase (OADC), 0.5% (v/v) glycerol, 0.05% (v/v) tyloxapol and hygromycin B (50 µg/mL). Bacteria were cultured in 50 mL cultures, in 250 mL vented Erlenmeyer flasks (Corning, Corning, NY, USA), for 7 days, at 37°C with shaking (200 rpm) to reach exponential growth phage (2).

Mycobacterium bovis BCG was cultured until an OD600 (optical density value at 600 nm) within the range of 0.6–1.0. 10 mL of bacterial culture was achieved. The desired volume of bacterial culture was washed with and resuspended in antibiotic-free cDMEM of an appropriate volume. BMDMs were seeded in 24-well plates the day before infection. Before infection, BMDMs were washed twice with antibiotic-free cDMEM. Next, 500 µL of bacteria-containing medium was added to each well of BMDMs. After 4 h of incubation, BMDMs were washed with PBS to remove

extracellular bacteria, and 1 mL of antibiotic-containing cDMEM was added to each well for prolonged incubation until samples were harvested at the indicated time points.

BMDM migration assay

Each well of a cell invasion and migration (CIM) plate is composed of an upper chamber and a lower chamber, separated by a microporous membrane. Macrophages added to the upper chamber were allowed to migrate through the membrane to the chemokine-containing lower chamber. As cells passed through the membrane they adhered to the underside of the membrane, which was embedded with electrodes. This generated a signal of electrical impedance and was reflected as an arbitrary unit (cell index) as shown in the migration trace. It has been confirmed that rising cell impedance correlates with an increasing number of migrated cells (3).

BMDM phagocytosis assay

First, 1×10^5 BMDMs were plated in a 96-well optical plate with black walls (Thermo Fisher) one day before the experiment. The next day, one vial of pHrodoTM Green Zymosan Bioparticles (Thermo Fisher) was thawed in 4 mL of Live Cell Imaging Solution (Thermo Fisher), and sonicated for 5 min on ice. The culture medium of the BMDMs was removed and replaced with 100 µL of bioparticle suspension. BMDMs in the control wells were incubated with bioparticle-free Live Cell Imaging Solution. The plate was transferred to an incubator at 37 °C. At each time point, cells were washed with PBS twice to remove extracellular bioparticles before fluorescence intensity was read using a Clariostar Microplate Reader (BMG LABTECH). Phagocytic activity was indicated by relative fluorescence activity, which was calculated by subtracting the fluorescence intensity of the particle-free control well from value of the corresponding experimental well.

Flow cytometry

Murine tissues were cut, crushed and filtered with a 70 µm cell strainer (Falcon). The resultant single cell suspensions were washed and resuspended in FACS buffer. Immunostaining was performed in a 96-well V-bottom plate (Corning). Splenocytes were used for single-colour staining for fluorescence compensation during data analysis. Cells were washed with FACS buffer twice and blocked with anti-mouse CD16/32 antibody (BioLegend) for 10 min at RT. When staining cell surface antigens, samples were incubated with a master mix of fluorophore-conjugated antibodies

for 30 min at 4 °C. After one wash with FACS buffer, samples were fixed by incubation with fixation buffer (BioLegend) for 10 min at RT followed by FACS wash. When intracellular staining (ICS) was also required, cells that were already stained with required cell surface antigens were fixed as described above, washed twice with 1× Permeabilisation Wash Buffer (BioLegend), and incubated with fluorophore-conjugated antibodies diluted in 1× Permeablisation Wash Buffer for 20 min in the dark at RT. Stained cells were washed and resuspended in FACS buffer for analysis. To detect nuclear FOXP3, samples that were already stained with required surface antigens were incubated with Fixation Permeabilisation Buffer (Thermo Fisher) for 30 min at RT. Samples were then washed with Permeabilisation Buffer (Thermo Fisher) twice followed by incubation with anti-FOXP3 antibody diluted in Permeabilisation Buffer for 30 min in the dark at RT. Next, cells were washed and resuspended in FACS buffer. A live cell fixable dye (L10119, Thermo Fisher) was used in all staining reactions to exclude dead cells. All of the centrifugation steps were performed at 350 g at 4 °C. Samples were analysed using an LSRFortessa X-20 cell analyser (BD Biosciences). FACS data were analysed using FlowJo v10.

Protein analysis

Adherent cells were lysed in tissue culture plates whereas mouse tissues were snap freezed, meshed, and lysed in Eppendorf tubes. Samples were lysed with urea buffer and supernatants were collected after centrifuging at 15,000 g for 15 min at 4°C . Protein concentration was determined using the Bio-Rad Bradford protein assay (Bio-Rad). Cell lysate of known volume was mixed with an appropriate volume of 6× Laemmli buffer. Samples were boiled for 10 min at 95 °C and were then ready for gel electrophoresis.

SDS-PAGE gels were prepared with a Mini-PROTEAN® Tetra Cell system (Bio-Rad or pre-cast 26-well Midi Protein Gels (Thermo Fisher). Prepared samples of equal amounts of protein and a pre-stained protein marker (New England Biolabs) were loaded into SDS-PAGE gels. Protein samples separated by SDS-PAGE were transferred onto a nitrocellulose membrane (Whatman) using a wet transfer system (Hoefer) loaded with 1× transfer buffer. The transfer was performed at a constant voltage of 80 V at 4 °C for 3 hours. The membrane was then blocked with 5% (w/v) milk (Marvel) in 1× TBST solution for 1 hour at room temperature before being incubated with primary antibody

diluted in 5% (w/v) milk or 5% (w/v) BSA overnight at 4°C. The membrane was washed with 1× TBST for 20 min and incubated with HRP-conjugated secondary antibody at room temperature for 1 hour. Next, the membrane was washed with 1× TBST for 20 min followed by the application of enhanced chemiluminescence (ECL) Western blotting detection reagent (GE Healthcare). The result was visualised with X-ray film (Fujifilm) using a film developer in the dark. If another antibody was required for immunoblotting, the membrane was incubated with stripping buffer for 20 min at 55°C and blocked again before application of the new primary antibody.

RNAi

siRNA oligos against mouse FIH, HIF1α, and HIF2α were purchased from Dharmacon. Sequences are available from Dharmacon or on request. We used siGENOME RISC-Free siRNA (Dharmacon) as a negative control. J774 cells were transfected with the indicated siRNA oligos at a final concentration of 20 nM using Dharmafect 1 reagent (Dharmacon) according to the manufacturer's instructions.

RNA extraction, reverse transcription, and real time-quantitative PCR (RT-qPCR)

RNA was extracted from cells using an RNeasy Mini Kit (QIAGEN) following the manufacturer's instructions. First, 200 ng of total RNA was used to prepare cDNA with the SuperScript III First-Strand Synthesis System (Thermo Fisher), according to the manufacturer's protocol. Next, 1 unit volume of cDNA sample obtained from reverse transcription was diluted by 4 unit volumes of RNase-free water. Each qPCR reaction was performed in duplicate using 2 µL of cDNA template, 2 µL of RNase-free water, 1 µL of primer mix at a final concentration of 100 nM, and 5 µL of SYBR green (QIAGEN). Quantitative PCR was performed using the gene-specific primers listed in Supplementary Table 8. StepOnePlus Real-Time PCR System was used to conduct the following thermal cycles: 95°C (10 min); 40 cycles of 94°C (15 s), -55°C (30 s), and -72°C (1 min); followed by the melting curve run. Data were collected at the elongation phase of each cycle. All the primers used were tested for amplification efficiency. The expression level of each target gene was analysed based on the ΔΔCt method with *Actg* as a reference gene. For genes that were not expressed in the control sample, the expression level was expressed as relative expression normalised to *Actg*.

RNA sequencing analysis

Generation of double stranded cDNA and library construction were performed using TruSeq® Stranded mRNA HT (RS-122-2103) with minor modifications to manufacturer specifications. The following custom primers (25 µM each) were used for the PCR enrichment step: multiplex PCR primer 1.0: 5'-AATGATACGGCGACCACCGAGATCTACACTCTTTCCCTACACGACGCTCTTCCGATCT-3'; Index primer: 5'-CAAGCAGAAGACGGCATACGAGAT[INDEX]CAGTGACTGGAGTTCAGACGTGTGCTCTTCCGATCT-3'. Indices were according to the eight bases tags developed by the Wellcome Centre for Human Genetics, University of Oxford (4). Amplified libraries were analysed for size distribution using the Agilent Tapestation 2200 D1000. Libraries were quantified using Picogreen and relative volumes were pooled accordingly. Sequencing was performed paired end read on a HiSeq4000 according to Illumina specifications with a read length of 125 bp. RNA seq reads were aligned to the mouse reference genome (GRCm38.85) using STAR and quantified by FeatureCount. The edgeR package (5) (version 3.26.1) from Bioconductor (version 3.1) was used to analyse the RNA sequencing data. Genes with at least one count per million in at least 4 samples were kept for downstream analysis. Gene expression in terms of raw counts was normalized using the trimmed means of M values (TMM) method, implemented by the calNormFactors function. Differential expression analysis was performed using the exactTest function and a gene was considered to be differentially expressed between $FIH^{+/+}$ and $FIH^{\Delta 1\text{-}2/\Delta 1\text{-}2}$ if absolute log2 fold change was > 1 and FDR was < 0.05. The resulting differentially expressed genes (DEGs) were visualized as heatmaps using R package pheatmap (6) (version 1.0.12). The R script can be accessed via https://zenodo.org/record/7339349#.Y3oot-xBxBw.

Statistical analysis

Statistical analysis was formed in GraphPad. Differences were considered significant at $p<0.05$. The Mantel–Cox test was conducted to determine the statistical significance of difference in spontaneous tumorigenesis between indicated mouse strains. The χ^2 test was used to compare the tumor incidence of mice of different genotypes. Grubbs' test was employed to identify outliers

in the datasets. A two-tailed, unpaired *t*-test was used to compare sizes of tumors at each time point, and data analysis of flow cytometry and RT-qPCR experiments, before which sample data were checked for normal distribution. The statistical tests used in each experiment are described within the relevant section of text.

SI References

1. I. R. Humphreys, *et al.*, A role for dendritic cells in the dissemination of mycobacterial infection. *Microbes Infect* **8**, 1339–46 (2006).

2. P. Bettencourt, *et al.*, Identification of antigens presented by MHC for vaccines against tuberculosis. *NPJ Vaccines* **5**, 2 (2020).

3. A. J. Iqbal, *et al.*, A real time chemotaxis assay unveils unique migratory profiles amongst different primary murine macrophages. *PLoS One* **8**, e58744 (2013).

4. S. Lamble, *et al.*, Improved workflows for high throughput library preparation using the transposome-based nextera system. *BMC Biotechnology* **13**, 104 (2013).

5. M. D. Robinson, D. J. McCarthy, G. K. Smyth, edgeR: a Bioconductor package for differential expression analysis of digital gene expression data. *Bioinformatics* **26**, 139–40 (2010).

6. R. Kolde, Pheatmap: pretty heatmaps. *R package version* **1**, 726 (2012).

Acknowledgments

Several cell lines and reagents used in this study were generous gifts from Drs. N Hogg, C Beisswenger, R Muschel, and R Reljic. We thank Professors Siamon Gordon, Colin Goding, and Richard Cornall for helpful discussions. We thank Richard Lisle and Robbie Crickley (Ludwig Institute for Cancer Research) for their assistance with imaging. This project was primarily supported by the Ludwig Institute for Cancer Research Ltd. D.R.G is funded by British Heart Foundation grant RG/15/10/31485.

Supplementary Table 1. Spectrum of spontaneous tumourigenesis in FIH$^{+/+}$, FIH$^{+/\Delta1\text{-}2}$, and FIH$^{\Delta 1\text{-}2/\Delta 1\text{-}2}$ mice

Code	Sex	Genotype	Age (weeks)	Type of tumour	Lymph node	Thymus	Spleen	Lung	Liver	Kidney	Peri-ureter fat	Peri-cardial fat
M00047067	F	FIH$^{+/+}$	122	B cell lymphoma	high grade		high grade					
M00047321	F	FIH$^{+/+}$	123.9	FDCS+B cell lymphoma			FDCS				high grade	
M00047087	F	FIH$^{+/+}$	88.4	B cell lymphoma			low grade				low grade	
XCFW5.3b	M	FIH$^{+/+}$	98	B cell lymphoma			high grade			high grade		
M00047032	F	FIH$^{+/\Delta1\text{-}2}$	59	B cell lymphoma (marginal zone lymphoma with components transforming to high grade)	low+high grade		low+high grade	low+high grade		low+high grade		
XCGW33.1d	F	FIH$^{+/\Delta1\text{-}2}$	64.5	B cell lymphoma (marginal zone lymphoma)	low grade		low grade					
M00047018	F	FIH$^{+/\Delta1\text{-}2}$	65	Myeloid leukaemia			AML					
M00047017	F	FIH$^{+/\Delta1\text{-}2}$	65	B cell lymphoma			high grade	high grade	high grade	high grade		
M00047027	M	FIH$^{+/\Delta1\text{-}2}$	71	B cell lymphoma			Low+high	low grade				low grade
M00037361	F	FIH$^{+/\Delta1\text{-}2}$	83	B cell lymphoma		low grade	high grade	high grade			low +high grade	low grade

Code	Sex	Genotype	Age (weeks)	Type of tumour	Lymph node	Thymus	Spleen	Lung	Liver	Kidney	Peri-ureter fat	Peri-cardial fat
M00037315	F	$FIH^{+/\Delta 1-2}$	86	B cell lymphoma (marginal zone lymphoma)				low grade				low grade
M00047089	F	$FIH^{+/\Delta 1-2}$	89	T cell lymphoma		T cell lymphoma	T cell lymphoma	T cell lymphoma	T cell lymphoma			
M00047293	F	$FIH^{+/\Delta 1-2}$	102	B cell lymphoma+mesothelioma		high grade			high grade			
M00047085	M	$FIH^{+/\Delta 1-2}$	104	Myeloid leukaemia			AML	AML	AML	AML		
M00047038	F	$FIH^{+/\Delta 1-2}$	113	B cell lymphoma	high grade		low +high grade					
M00047016	F	$FIH^{+/\Delta 1-2}$	116.9	B cell lymphoma (marginal zone lymphoma with components transforming to high grade) +colon adenocarcinoma	low+high		low grade	low grade		low grade		
M00047019	F	$FIH^{+/\Delta 1-2}$	116.9	B cell lymphoma			high grade	Low grade				
M00037084	F	$FIH^{\Delta 1-2/\Delta 1-2}$	95	B cell lymphoma (marginal zone lymphoma with components			low+high grade	low grade				

Code	Sex	Genotype	Age (weeks)	Type of tumour	Lymph node	Thymus	Spleen	Lung	Liver	Kidney	Peri-ureter fat	Peri-cardial fat
				transforming to high grade), HCC								
M00047291	F	$FIH^{\Delta1\text{-}2/\Delta1\text{-}2}$	107	B cell lymphoma (marginal zone lymphoma)	low grade		low grade	low grade	low grade	low grade		low grade
XCGW14.3c	F	$FIH^{\Delta1\text{-}2/\Delta1\text{-}2}$	68	B cell lymphoma			low grade	high grade				
XCGW34.5d	F	$FIH^{\Delta1\text{-}2/\Delta1\text{-}2}$	13	B cell lymphoma			low grade	low grade		low grade	low grade	
XCGW15.1a	F	$FIH^{\Delta1\text{-}2/\Delta1\text{-}2}$	74	B cell lymphoma (marginal zone lymphoma)			low grade	low grade			low grade	
M00037083	F	$FIH^{\Delta1\text{-}2/\Delta1\text{-}2}$	109	B cell lymphoma (marginal zone lymphoma)			low grade	low grade		low grade		low grade
M00047322	F	$FIH^{\Delta1\text{-}2/\Delta1\text{-}2}$	83	B cell lymphoma (marginal zone lymphoma with components transforming to high grade)			low+high grade	low grade	low grade	high grade		
XCGW15.1b	F	$FIH^{\Delta1\text{-}2/\Delta1\text{-}2}$	104	B cell lymphoma (marginal zone lymphoma with components	high grade		low+high grade					

Code	Sex	Genotype	Age (weeks)	Type of tumour	Lymph node	Thymus	Spleen	Lung	Liver	Kidney	Peri-ureter fat	Peri-cardial fat
				transforming to high grade)								
XCGW14.3d	F	$FIH^{\Delta1-2/\Delta1-2}$	97.8	B cell lymphoma (marginal zone lymphoma)			low grade	low grade		low grade		
XCFW5.1f	F	$FIH^{\Delta1-2/\Delta1-2}$	88.8	B cell lymphoma			low grade					
XCGW28.2e	F	$FIH^{\Delta1-2/\Delta1-2}$	83.8	B cell lymphoma (marginal zone lymphoma)			low+high grade	low grade				
XCFW3.4g	M	$FIH^{\Delta1-2/\Delta1-2}$	88.6	B cell lymphoma			low grade	low grade				
XCFW3.3c	M	$FIH^{\Delta1-2/\Delta1-2}$	92	B cell lymphoma			low+high grade	low grade				
XCFW3.4a	M	$FIH^{\Delta1-2/\Delta1-2}$	105.8	Lung adenocarcinoma								
XCFW3.4b	M	$FIH^{\Delta1-2/\Delta1-2}$	88.6	B cell lymphoma (marginal zone lymphoma)			low grade	low grade		low grade	low grade	

FDCS: follicular dendritic cell sarcoma

AML: acute myeloid leukaemia

HCC: hepatocellular carcinoma

DLBCL: diffuse large B-cell lymphoma

Supplementary Table 2. LLC tumour volumes in FIH$^{+/+}$, FIH$^{+/\Delta 1\text{-}2}$, and FIH$^{\Delta 1\text{-}2/\Delta 1\text{-}2}$ mice

Genotype/tumour volume (mm^3)

Time point	FIH$^{+/+}$									
D10	88.48	87.64	34.46		137.31	136.46	115.20	47.07	75.12	25.60
D12	138.92	127.83	45.56	146.85	145.93	139.26	196.00	52.27	136.80	54.68
D13	191.10	163.37	51.91	174.24	201.64	140.98	196.52	103.68	139.97	58.24
D14	232.73	156.28	62.50	196.00	213.16	153.76	248.37	133.75	157.60	62.96

Time point	FIH$^{+/\Delta 1\text{-}2}$													
D10	108.84	71.25	201.14	129.96	154.42	131.20	111.39	81.47	173.03	138.38	254.95	124.47	147.46	134.14
D12	172.83	109.65	228.10	245.48	207.36	196.00	180.34	242.76	178.18	198.45	264.38	169.98	273.78	215.04
D13	213.16	128.77	385.78	316.37	240.10	369.80	240.12	213.15	265.88	355.74	564.54	231.89	326.11	352.00
D14	263.84	185.68	496.86	397.38	259.20	376.54	303.12	208.25	277.26	377.50	773.66	403.44	461.29	469.80

Time point	FIH$^{\Delta 1\text{-}2/\Delta 1\text{-}2}$			
D10	90.75	166.70	100.72	135.00
D12	143.36	213.15	185.02	208.09
D13	163.84	267.19	292.38	251.26
D14	212.54	334.61	518.94	312.67

	No. tumour measured		
Time point	FIH$^{+/+}$	FIH$^{+/\Delta 1\text{-}2}$	FIH$^{\Delta 1\text{-}2/\Delta 1\text{-}2}$
D10	9	14	4
D12	10	14	4
D13	10	14	4
D14	10	14	4

	No. mice with tumour		
Time point	FIH$^{+/+}$	FIH$^{+/\Delta 1\text{-}2}$	FIH$^{\Delta 1\text{-}2/\Delta 1\text{-}2}$
D10	5	7	2
D12	5	7	2
D13	5	7	2
D14	5	7	2

	Mean tumour volume (mm^3			P value	
Time point	FIH$^{+/+}$	FIH$^{+/\Delta 1\text{-}2}$	FIH$^{\Delta 1\text{-}2/\Delta 1\text{-}2}$	FIH$^{+/+}$ vs FIH$^{+/\Delta 1\text{-}2}$	FIH$^{+/+}$ vs FIH$^{\Delta 1\text{-}2/\Delta 1\text{-}2}$
D10	83.038	140.1	123.292	0.0073	0.121
D12	118.41	205.9	187.405	0.0002	0.027
D13	142.17	300.2	243.665	0.0003	0.009
D14	161.71	375.3	344.69	0.0004	0.003

Supplementary Table 3. B16 tumour volumes in FIH$^{+/+}$, FIH$^{+/\Delta1\text{-}2}$, and FIH$^{\Delta1\text{-}2/\Delta1\text{-}2}$ mice

	Genotype/tumour volume (mm^3)																					
Time point	FIH$^{+/+}$								FIH$^{+/\Delta1\text{-}2}$								FIH$^{\Delta1\text{-}2/\Delta1\text{-}2}$					
D8	6.91	23.04	4.63	9.84	6.91	6.08	58.73	13.50	43.32	9.84	32.00	45.56	69.34			44.98	16.38	24.06	37.04	98.32	32.00	50.40
D9	10.98	25.60	16.38	19.65	12.19	8.79	95.51	27.56	61.06	21.96	87.50	112.50	58.24			113.72	32.00	49.92	39.75	196*	50.625*	67.84
D10	19.65	44.00	32.00	53.96	27.44	17.97	108.00	43.42	76.95	54.68	94.64	188.53	175.34	55.30		158.76*	32.00	88.48	74.44			105.88
D11	21.44	63.89	45.56	51.91	60.75	27.44	169.00	87.50	121.68	65.81	137.3125*	276.85*	254.32	137.31	17.97		67.24	143.75	108.00			220.50
D12	25.33	83.75	96.80	99.09	96.25	78.75	202.61	100.00	214.33	116.64			276.85	219.49	196.00		104.10	215.60	256.00			405.00
D14	55.69	202.01	246.24	304.00	192.20	157.60	171.50	126.85	323.46	163.37			259.84	275.68	384.75		230.64	423.20	289.13			624.24

**=mouse culled due to tumour ulceration*

	No. tumour measured		
Time point	FIH$^{+/+}$	FIH$^{+/\Delta1\text{-}2}$	FIH$^{\Delta1\text{-}2/\Delta1\text{-}2}$
D8	8	6	6
D9	8	6	6
D10	8	7	4
D11	8	7	4
D12	8	5	4
D14	8	5	4

	No. mice with tumour		
Time point	FIH$^{+/+}$	FIH$^{+/\Delta1\text{-}2}$	FIH$^{\Delta1\text{-}2/\Delta1\text{-}2}$
D8	5	5	4
D9	5	5	4
D10	5	6	3
D11	5	6	3
D12	5	5	3
D14	5	5	3

	Mean tumour volume (mm^3)			P-value	
Time point	FIH$^{+/+}$	FIH$^{+/\Delta1\text{-}2}$	FIH$^{\Delta1\text{-}2/\Delta1\text{-}2}$	FIH$^{+/+}$ vs FIH$^{+/\Delta1\text{-}2}$	FIH$^{+/+}$ vs FIH$^{\Delta1\text{-}2/\Delta1\text{-}2}$
D8	16.21	40.84	43.03	0.0314	0.0565
D9	27.08	75.83	72.69	0.0146	0.0870
D10	43.3	114.9	75.2	0.0085	0.1108
D11	65.94	144.5	134.9	0.0554	0.0589
D12	97.82	204.7	245.2	0.0043	0.0128
D14	182	281.4	391.8	0.0459	0.0134

Supplementary Table 4. LLC tumour volumes in WT and myeloid-specific FIH knockout (FIH MKO) mice

Time point	Genotype/tumour volume (mm^3)																										
	WT																										
D7	44.00	29.60	52.92	65.81							33.60	20.25	33.21	35.28	47.63	92.60	32.00	13.75	33.69	32.40							
D8	50.27	43.56	40.84	87.50	54.15						45.50	23.33	41.60	45.18	62.50	100.92	31.21	22.53	55.47	63.48							
D9	48.51	41.45	47.30	85.18	60.84	25.94	106.25	50.34	118.98	88.70	38.29	20.42	37.13	39.45	44.17	74.62	54.21	22.82	51.54	54.08	63.36	46.23	90.40	73.96	48.40		
D10	46.40	36.75	46.40	138.60	60.00	47.78	123.60	81.12	112.50	90.0375*	72.83	40.00	70.00	60.52	87.81	144.87	94.94	37.54	71.94	80.00	87.50	77.25	87.50	66.15	90.94		
D12	77.50	61.02	87.83	112.36	47.91	58.40															113.72	86.21	113.49	81.00	92.51		
D13	84.53	68.73	97.34	153.00	58.73	74.93	190.13	87.08	247.01		118.80	43.20	133.20	204.66	120.60	254.63	129.21	84.27	146.21	87.50	126.00	115.35	157.22	106.25	117.74		
	FIH MKO																										
D7		62.50	51.91	54.68							75.00	25.59	60.02	24.50	42.88	28.16	60.80	64.72	32.46	42.40	31.50						
D8		62.50	67.76	64.72							100.92	58.75	108.16	48.00	62.40	43.32	95.00	81.00	43.81	52.90	52.27						
D9	50.34	53.66	62.78	61.95	105.00	133.10	129.96	135.20	116.25	116.27	67.28	45.83	85.72	35.10	64.17	47.86	73.87	66.67	32.00	41.97	40.66	59.90	92.70	93.78	132.54	96.19	81.00
D10	74.44	67.50	88.75	87.50	121.68	122.47	166.70	174.05	135.20	154.79	119.16	67.23	122.47	86.40	125.54	77.23	196*	112.50	46.55	82.84	86.44	147.46	137.68	144.87	115.56	253.50	88.87
D12	81.12	87.50	97.34	104.98																		168.3375*	152.10	222.376 *	182.52	289*	99.41
D13	129.60	129.96	131.22	124.47	117.62	295.70	269.57	294.00	303.75	224.45	210.83	135.00	117.98	180.00	194.35	142.96		192.60	92.46	116.46	109.35		204.91		129.43		118.75

**mouse culled due to tumour ulceration*

Time point	No. tumour measured	
	WT	FIH MKO
D7	14	14
D8	15	14
D9	25	27
D10	25	27
D12	11	10
D13	24	23

Time point	No. mice with measurable tumours	
	WT	FIH MKO
D7	14	14
D8	15	14
D9	25	27
D10	25	27
D12	11	10
D13	24	23

Time point	Mean tumour volume (mm^3)		
	WT	FIH MKO	P value
D7	40.48	43.81	0.6577
D8	51.2	62.77	0.2020
D9	57.3	78.58	0.0117
D10	78.12	118.6	0.0004
D12	84.72	148.5	0.0086
D13	125.3	172.4	0.0107

Supplementary Table 5. B16 tumour volumes in WT and FIH MKO mice

	Genotype/tumour volume (mm^3)																		
**Time point**	**WT**											**FIH MKO**							
D10	27.44	7.81	146.85	0.00	78.73	0.00	0.00	70.88	60.03	19.65	13.50	78.73	90.00	40.43	95.00	268.36	202.61	167.94	43.20
D11	42.59	37.04	97.56	210.94	175.07	59.05	179.56	116.96	158.76	32.00	62.50	133.12	256.00	115.17	133.43	220.32	194.35	180.00	95.99
D13	219.70	55.23	32.00	271.47	203.35	113.49	386.63	290.52	255.94	81.93	117.98	438.89	564.54	112.36	198.45	443.76	243.68	372.60	319.74

	No.tumour	
**Time point**	**WT**	**FIH MKO**
D10	3	8
D11	8	8
D13	8	8

	No. Mice	
**Time point**	**WT**	**FIH MKO**
D10	2	4
D11	5	4
D13	5	4

	Mean tumour volume (mm^3)		
**Time point**	**FIH WT**	**FIH MKO**	**P value**
D10	53.0562	123.3	0.0528
D11	106.586	166	0.0526
D13	184.412	336.8	0.0205

Supplementary Table 6. LLC tumour volumes in WT and HIF1a MKO mice

	Genotype/tumour volume (mm^3)																										
**Time point**	**WT**																										
D7	32.40	49.00	55.20	88.70	62.96	62.96	38.40	32.46	41.60	13.50	32.67	45.36	59.09		31.58	38.27	66.82	35.84	32.17								
D8	35.39	76.73	35.84	106.74	113.72	84.53	44.55	52.92	47.59	32.00	49.28	49.28	67.49		33.69	41.15	61.36	48.00	41.07	28.88	83.11	88.94	70.23	70.88	36.00	44.06	56.00
D11	100.00	200.69	73.57	174.64	151.38	160.00	117.98	86.15	97.47	66.83	65.60	112.90	116.64		39.71	87.50	98.32	125.44	63.89								
D12																				88.36	115.20	240.12	246.40	178.18	83.82	172.98	176.75
D14	250.47	443.76	180.67	396.12	346.85	314.24	213.15	200.38	172.83	151.38	162.00	221.81	201.81	16.46	241.88	142.97	83.75	196.04	98.31	118.98					126.51		
	**HIF1a MKO**																										
D7	59.17	67.24	74.93	37.04	58.19	31.79	70.69																				
D8	97.34	75.65	93.75	39.69	81.12	36.10	60.02	37.60	20.18	33.70	95.29	49.92	42.53	41.15													
D11	141.33	186.05	153.00	88.48	131.20	55.47	102.74																				
D12								169.00	74.44		247.80	147.99	107.65	113.72													
D14	269.50	355.74	389.80	127.05	205.80	120.21	109.35		105.97																		

	No.tumour measured	
**Time point**	**WT**	**HIF1a MKO**
D7	18	7
D8	26	14
D11	18	7
D12	8	6
D14	21	8

	No. Mice with measurable tumour	
**Time point**	**WT**	**HIF1a MKO**
D7	18	7
D8	26	14
D11	18	7
D12	8	6
D14	21	8

	Mean tumour volume (mm^3)		
**Time point**	**WT**	**HIF1a MKO**	**P value**
D7	45.5	57.01	0.15187
D8	57.67	59.26	0.848395
D11	107.7	122.6	0.441644
D12	162.7	143.4	0.574739
D14	203.8	210.4	0.882822

Supplementary Table 7. LLC tumour volumes in WT, HIF2a MKO, FIH HIF2a myeloid double knockout (MDKO) mice

	Genotype/tumour volume (mm^3)																						
Time point	WT																						
D7	57.32	13.50	19.32	45.60	48.00		12.38	49.28		45.63	21.78	25.05	92.16	48.00	65.81		26.68						
D9	74.54	21.44	48.00	108.90	131.22						48.00	51.20	162.45	61.85	142.96	13.50	85.00						
D11	116.46	30.12	71.79	121.84	146.21	122.19	56.80	115.17	9.84	53.62													
D14	235.67	78.42			326.10	224.45	97.34	217.09	42.59	109.76	176.61	121.84		136.80	331.88	40.00	113.44						
	HIF2a MKO																						
D7	8.79	6.91		24.58		13.50		55.30	16.65	30.63	36.50	41.15	16.38	25.33		48.00	17.10	46.40	39.20	19.80	21.50		40.43
D9	32.00	17.97	10.00	64.86	13.50	21.44	20.25	71.25								56.00	57.71	65.81	98.32	45.86	53.24	24.75	69.63
D11	40.00	27.22	32.40	99.14	29.48	13.72	40.00	99.09	56.07	149.92	76.83	90.00	57.43	59.90									
D14	80.64	74.36	75.94	153.76	52.92	44.10	66.27	137.22	97.47				87.48		17.97	149.45			196.00	98.32	134.54	54.68	133.96
	FIH HIF2a DKO																						
D7	34.85	7.81	33.52	36.00		75.00		45.94	22.87	35.20	58.08	64.98	58.40	23.41	25.92	33.93	20.48						
D9											94.64	115.00	85.05	14.90	20.48	65.17	32.00						
D11	113.44	28.86	95.22	46.23	26.01	153.16	30.12	69.70	45.56	101.92													
D14	182.75	45.60		63.79	34.23		66.25	107.39	40.57	156.82				75.00	164.78	100.00	81.25						

	No.tumour measured		
Time point	WT	HIF2a MKO	FIH HIF2a MDKO
D7	14	18	16
D9	12	16	7
D11	10	14	10
D14	14	17	12

	No. Mice with measurable tumour		
Time point	WT	HIF2a MKO	FIH HIF2a MDKO
D7	14	18	16
D9	12	16	7
D11	10	14	10
D14	14	17	12

	Mean tumour volume (mm^3)			P value		
Time point	WT	HIF2a MKO	FIH HIF2a MDKO	WT vs HIF2a MDKO	FIH MKO vs FIH HIF2a MDKO	WT vs HIF2a FIH MDKO
D7	40.75	28.23	38.43	0.0655	0.0861	0.7634
D9	79.09	45.16	61.03	0.0233	0.2605	0.4116
D11	84.4	62.23	71.02	0.2050	0.5989	0.5109
D14	160.9	97.36	93.2	0.0212	0.8215	0.0361

Supplementary Table 8. List of primers for RT-qPCR

Target gene	Designed primers (5’-3’)	
Actg	F	CCAACAGCAGACTTCCAGGATT
	R	CTGGCAAGAAGGAGTGGTAACTG
Nos2	F	TGGTGAAGGGACTGAGCTGT
	R	CTGAGAACAGCACAAGGGGT

Target gene	Primers purchased from Qiagen
Arg1	QT00134288
Hif1an	QT00133035
Il10	QT00106169
Il6	QT00098875
Tnfa	QT00104006
Vegfa	QT00160769
Tgfb1	QT249900

Supplementary Table 9. Antibodies used for IHC/ICC/WB
Primary antibodies

Antigen	Clone	Host	Dilution	Source	Application
ARG1	-	Rabbit	1 in 2000	abcam (ab91279)	WB, IHC
ARG1	-	Rabbit	1 in 1000	Abcam (ab 96183)	IF, IHC
B220	RA3-6B2	Rat	1 in 300	BD 550286	IHC
CD4	-	Rabbit	1 in 200	Bioss (bs-0766R)	IHC
CD8	-	Rabbit	1 in 200	Bioss (bs-0648R)	IHC
F4/80	CI: A3-1	Rat	1 in 500	Bio-Rad MCA497G	IF, IHC
FIH	3F9	Mouse	1 in 25	Moravian Biotech	WB
FOXP3	FJK-16s	Rat	1 in 50	Thermo Fisher (11-5773-82)	IHC
HIF-2α	-	Rabbit	1 in 3000	Ratcliffe Lab	IHC
NOS2	-	Rabbit	1 in 2000	Millipore (06-573)	WB, IHC
β-tubulin	-	Rabbit	1 in 4000	abcam (ab6046)	WB
VEGFA	-	Rabbit	1 in 2000	ThermoFisher 26157-1-AP	IHC

Supplementary Table 10. Antibodies used for flow cytometry

Antigen	Clone	Fluorochrome	Usage/ test (μL)	Source
ARG1	-	FITC	1.0	R&D Systems (IC5868F)
B220	RA3-6B2	PB	0.5	BioLegend (103230)
B220	RA3-6B2	PE/Cy7	0.25	BioLegend (103221)
C5AR	20/70	APC	0.5	BioLegend (135807)
CC5R	HM-CCR5	PE	0.5	BioLegend (107005)
CD11b	M1/70	FITC	0.25	Thermo Fisher (11-0112-82)
CD11b	M1/70	PerCP/Cy5.5	0.25	BD (561114)
CD11c	HL3	PE/Cy7	0.25	BD (561022)
CD25	PC61	PB	0.5	BioLegend (102021)
CD4	GK1.5	BV421	0.25	BioLegend (100437)
CD4	RM4-4	FITC	0.5	Thermo Fisher (11-0043-82)
CD49b	DX5	PE	0.5	BioLegend (108907)
TCRγδ	GL3	APC	0.5	BioLegend (111205)
CD8a	53-6.7	BV510	0.5	BioLegend (100751)
CD8a	53-6.7	APC	0.5	Thermo Fisher (17-0081-81)
F4/80	BM8	Pacific Blue	0.25	BioLegend (123123)
F4/80	BM8	PerCP/Cy5.5	0.25	BioLegend (123127)
FOXP3	150D	PE	0.25	BioLegend (320007)
Ly-6C	HK1.4	APC	0.25	BioLegend (128015)
Ly-6G	1A8	PE	0.25	BD (561104)
NK1.1	PK136	FITC	0.25	Thermo Fisher (11-5941-81)

FITC: Fluorescein Isothiocyanate; PE: Phycoerythrin; APC: Allophycocyanin; PerCP/Cy5.5: Peridinin-chlorophyll protein/Cyanine5.5; PE/Cy7: Phycoerythrin/Cyanine7; BV421: Brilliant Violet 421; BV510: Brilliant Violet 510; BD: BD Biosciences.

Supplementary Table 11. Cell lines

Cell line	Host	Source
L929	*Mus musculus*, fibroblast	Prof. N Hoggs, The Francis Crick Institute, UK
B16F10	*Mus musculus*, melanoma	ATCC
LLC	*Mus musculus*, lung carcinoma	Dr. C Beisswenger, Universitätsklinikum des Saarlandes, Germany
CT26	*Mus musculus*, colon adenocarcinoma	Prof. R Muschel, University of Oxford, UK
J774	*Mus musculus*, macrophages	Prof. Helen McShane, University of Oxford, UK

LLC: Lewis Lung Carcinoma; ATCC: American Type Culture Collection
